# Sex Differences in Mental Health Problems and Psychiatric Hospitalization in Autistic Young Adults

**DOI:** 10.1001/jamapsychiatry.2022.3475

**Published:** 2022-10-26

**Authors:** Miriam I. Martini, Ralf Kuja-Halkola, Agnieszka Butwicka, Ebba Du Rietz, Brian M. D’Onofrio, Francesca Happé, Aleksandra Kanina, Henrik Larsson, Sebastian Lundström, Joanna Martin, Mina A. Rosenqvist, Paul Lichtenstein, Mark J. Taylor

**Affiliations:** 1 Department of Medical Epidemiology and Biostatistics, Karolinska Institutet, Stockholm, Sweden; 2 Department of Child Psychiatry, Medical University of Warsaw, Warsaw, Poland; 3 Child and Adolescent Psychiatry, Stockholm Health Care Services, Region Stockholm, Sweden; 4 Department of Biostatistics and Translational Medicine, Medical University of Lodz, Lodz, Poland; 5 Department of Psychological and Brain Sciences, Indiana University, Bloomington; 6 Social, Genetic, and Developmental Psychiatry Centre, Institute of Psychiatry, Psychology, and Neuroscience, King’s College London, London, United Kingdom; 7 School of Medical Sciences, Örebro University, Örebro, Sweden; 8 Gillberg Neuropsychiatry Centre, University of Gothenburg, Gothenburg, Sweden; 9 Sweden Centre for Ethics, Law and Mental Health, University of Gothenburg, Gothenburg, Sweden; 10 MRC Centre for Neuropsychiatric Genetics and Genomics, Cardiff University, Cardiff, United Kingdom

## Abstract

**Importance:**

Psychiatric disorders are common among autistic children and adults. Little is known about sex differences in psychiatric disorders and hospitalization in early adulthood.

**Objective:**

To examine sex differences in psychiatric diagnoses and hospitalizations in autistic compared with nonautistic young adults.

**Design, Setting, and Participants:**

This population-based cohort study assessed all individuals born in Sweden between 1985 and 1997. A total of 1 335 753 individuals, including 20 841 autistic individuals (7129 [34.2%] female individuals), were followed up from age 16 through 24 years between 2001 and 2013. Analysis took place between June 2021 and August 2022.

**Exposures:**

Autism was defined as having received at least 1 clinical diagnosis of autism based on the *International Classification of Diseases*.

**Main Outcomes and Measures:**

The cumulative incidence of 11 psychiatric diagnoses up until age 25 years was estimated, and birth year–standardized risk difference was used to compare autistic female and male individuals directly. Sex-specific birth year–adjusted hazard ratios (HRs) with 95% CIs were calculated using Cox regression. Analyses were repeated for inpatient diagnoses to assess psychiatric hospitalization.

**Results:**

Of 1 335 753 individuals included in this study, 650 314 (48.7%) were assigned female at birth. Autism was clinically diagnosed in 20 841 individuals (1.6%; 7129 [34.2%] female) with a mean (SD) age of 16.1 (5.1) years (17.0 [4.8] years in female individuals and 15.7 [5.2] years in male individuals) for the first recorded autism diagnosis. For most disorders, autistic female individuals were at higher risk for psychiatric diagnoses and hospitalizations. By age 25 years, 77 of 100 autistic female individuals and 62 of 100 autistic male individuals received at least 1 psychiatric diagnosis. Statistically significant standardized risk differences were observed between autistic female and male individuals for any psychiatric disorder (−0.18; 95% CI, −0.26 to −0.10) and specifically for anxiety, depressive, and sleep disorders. Risk differences were larger among autistic than nonautistic individuals. Compared with nonautistic same-sex individuals, autistic female individuals (HR range [95% CI], 3.17 [2.50-4.04.]-20.78 [18.48-23.37]) and male individuals (HR range [95% CI], 2.98 [2.75-3.23]-18.52 [17.07-20.08]) were both at increased risk for all psychiatric diagnoses. Any psychiatric hospitalization was statistically significantly more common in autistic female individuals (32 of 100) compared with autistic male individuals (19 of 100). However, both autistic female and male individuals had a higher relative risk for psychiatric hospitalization compared with nonautistic female and male individuals for all disorders (female individuals: HR range [95% CI], 5.55 [4.63-6.66]-26.30 [21.50-32.16]; male individuals: HR range [95% CI], 3.79 [3.22-4.45]-29.36 [24.04-35.87]).

**Conclusions and Relevance:**

These findings highlight the need for profound mental health services among autistic young adults. Autistic female individuals, who experience more psychiatric difficulties at different levels of care, require increased clinical surveillance and support.

## Key Points

**Question:**

To what extent do young autistic female and male individuals differ in their psychiatric diagnoses and hospitalizations compared with nonautistic individuals?

**Findings:**

In this population-based cohort study, autistic individuals had higher cumulative incidences for psychiatric disorders and hospitalizations. Compared with autistic male individuals, autistic female individuals were more likely to receive diagnoses and be hospitalized for psychiatric disorders, particularly anxiety, sleep, and depressive disorders.

**Meaning:**

These findings show the high mental health needs of autistic young adults, particularly autistic female individuals who are at higher risk of receiving a diagnosis of, as well as being hospitalized for, psychiatric problems compared with autistic male individuals.

## Teaser

This cohort study examines sex differences in psychiatric diagnoses and hospitalizations in autistic compared with nonautistic young adults.

## Introduction

Mental health problems are a major concern in autistic individuals^1,2^ (note that we use identity-first language [autistic person] rather than person-first language [person with autism] throughout this article according to preferences reported by autistic individuals and their families^3^). Around 70% of autistic children meet diagnostic criteria for at least 1 psychiatric disorder,^4^ and 54%^5^ to 79%^6^ of autistic adults receive at least 1 psychiatric diagnosis. Mental health problems are reported even among autistic individuals showing good outcomes in other areas of functioning.^7^

Sex differences in mental health have been observed in the general population.^8^ (Note: The term *sex* refers to biological attributes of being female or male and is assigned at birth, whereas *gender* refers to socially constructed attributes^9^ as discussed in detail elsewhere.^10,11^) Yet, very few studies have directly investigated sex differences in psychiatric disorders among autistic individuals. Existing evidence suggests that autistic women are particularly vulnerable to psychiatric disorders^5^ and access psychiatric care more often than autistic men.^12^ One study^13^ on health care claims data of children and youth younger than 21 years suggested higher odds of psychiatric difficulties such as anxiety, mood, and sleep disorders in autistic girls compared with boys. A study^14^ using self-reported gender (instead of sex assigned at birth) in a smaller adult sample found that autistic women experience anxiety, depression, and eating disorders at higher rates than autistic men. To our knowledge, there is only 1 large population-based investigation^15^ of sex differences across mental health problems in a Danish cohort of autistic and nonautistic children and adolescents. This study showed that autistic female children and adolescents were at increased risk for several psychiatric disorders, compared with autistic male counterparts.

A limitation of the aforementioned study is that individuals were only followed up until age 16 years. Therefore, disorders with a later onset, specifically in young adulthood, were not covered. The median age at onset for psychiatric disorders was reported to be 18 years, and more than 60% of the psychiatric problems observed in adulthood emerge for the first time in the transitional period across adolescence and young adulthood before age 25 years.^16^ This highlights the importance of young adulthood as a particularly sensitive period when psychiatric disorders commonly develop, which is supported by a higher prevalence of psychiatric conditions reported in autistic compared with nonautistic transition-aged youth.^17^

Moreover, no study to date and to our knowledge has examined sex differences in psychiatric diagnoses at different levels of psychiatric care among autistic individuals. This is especially important given higher unmet health care needs in autistic individuals^18^ due to difficulties in accessing treatment, which might exacerbate mental health problems.^19^

Using representative data from Swedish population registers, we aimed to explore sex differences in psychiatric diagnoses and psychiatric hospitalization among autistic young adults compared with nonautistic individuals.

## Methods

### Study Population

This register-based cohort study was approved by the Regional Ethics Review Board in Stockholm and follows the Strengthening the Reporting of Observational Studies in Epidemiology (STROBE) reporting guideline. Register studies do not require informed consent in Sweden. The study was preregistered via the Open Science Framework on June 16, 2021 (https://doi.org/10.17605/OSF.IO/QZHJS).

We linked several nationwide Swedish registers (eAppendix in the Supplement). From the Medical Birth Register^20^ we identified all individuals born in Sweden between 1985 and 1997 (N = 1 407 253). The follow-up period was 2001 to 2013. We excluded individuals with chromosomal abnormalities (eTable 1 in the Supplement), stillbirths, individuals who died or migrated before their 16th birthday, and whose biological mother was unidentifiable. As data on gender were unavailable, we used sex assigned at birth (male/female) in the Medical Birth Register. We additionally identified sex using the Total Population Register^21^ and excluded individuals whose sex was missing or reported differently in the 2 registers, since we could not determine the cause of the mismatch or missing values. After exclusions, the eligible cohort included 1 335 753 individuals who were followed up from age 16 years until a diagnosis of the respective psychiatric disorder, their 25th birthday, death, emigration, or end of follow-up on December 31, 2013, whichever came first. The cohort selection process is depicted in eFigure 1 in the Supplement.

### Measures

We defined exposure as receiving at least 1 autism diagnosis from age 1 year onward based on *International Classification of Diseases *(*ICD*)*, Ninth Revision *code 299A and *ICD-10* codes F84 in the National Patient Register,^22^ excluding Rett syndrome, other childhood disintegrative disorders, and overactive disorder associated with intellectual disability (ID) and stereotyped movements. The validity of register-based autism diagnosis was reported to be high.^23^ Autism is a lifelong condition, and age at diagnosis is unlikely to accurately indicate age at onset. We therefore adopted a lifetime approach to autism (with sensitivity analyses to examine the impact of this approach). We defined psychiatric disorders as receiving a clinical outpatient or inpatient diagnosis in the National Patient Register between ages 16 and 24 years; diagnoses received before and after this age span were not considered. We looked at 11 individual disorders, recording the first occurrence of each diagnosis separately as well as first among all diagnoses (any diagnosis). For psychotic, bipolar, and sleep disorders we identified additional individuals through a dispensed prescription of medication in the Prescribed Drug Register^24,25^ (eTable 2 in the Supplement for *ICD* and Anatomical Therapeutic Chemical codes). Psychiatric hospitalization was assessed by considering only inpatient admission where psychiatric disorders were the primary reason for hospitalization.

Birth year, attention-deficit/hyperactivity disorder (ADHD), and ID were selected as covariates due to their strong associations with autism.^4,26,27^ ADHD and ID were identified based on a clinical diagnosis in the National Patient Register. Additional individuals with ADHD were identified through a prescription of ADHD medication in the Prescribed Drug Register (eTable 3 in the Supplement).

### Statistical Analyses

Analysis took place between June 2021 and August 2022. Data management was performed in SAS statistical software version 9.4.6 (SAS Institute). Data were analyzed using R version 4.0.5 (R Foundation) with the survival,^28^ drgee,^29^ and stdReg packages.^30^

Based on matching 10 nonautistic individuals to each autistic individual on sex and birth year, we calculated the sex-specific cumulative incidence at age 25 years as the proportion of individuals who received the respective diagnosis prior to that age, using Kaplan-Meier estimation and thus accounting for censoring during follow-up. To compare autistic female and male individuals, we calculated the birth year–standardized survival probability and risk difference. We further calculated standardized risk differences in nonautistic individuals for comparison. In the full sample, we then used sex-stratified Cox regression models to compare autistic and nonautistic individuals of the same sex while accounting for differences in follow-up time. We calculated hazard ratios (HRs) and 95% CIs for any as well as for each individual psychiatric diagnosis. Attained age was the underlying time scale. In all analyses, we fitted a crude model, a second model adjusted for birth year and a third model further adjusted for ADHD and ID. To explore sex differences in psychiatric hospitalization, we repeated all analyses using only inpatient diagnoses.

To account for multiple testing, we used Bonferroni-corrected significance levels in all analyses, adjusted for the number of psychiatric disorders investigated (any disorder and 11 individual psychiatric disorders). Two-sided *P* values were statistically significant at α = .004. For all models, CIs were estimated using a cluster robust sandwich estimator to account for related individuals in the sample using the maternal identity number.

### Sensitivity Analyses

To examine the impact of the lifetime approach to defining autism, we reran the analyses restricting autistic individuals to those diagnosed with autism before age 16 years (a total of 9747 individuals, of which 2731 [28.1%] were female). We performed another sensitivity analysis including only individuals who received an autism diagnosis on more than 1 occasion (a total of 15 735 individuals, of which 5460 [34.7%] were female) to account for diagnostic uncertainty.

## Results

### Cohort Description

**Figure 1.**
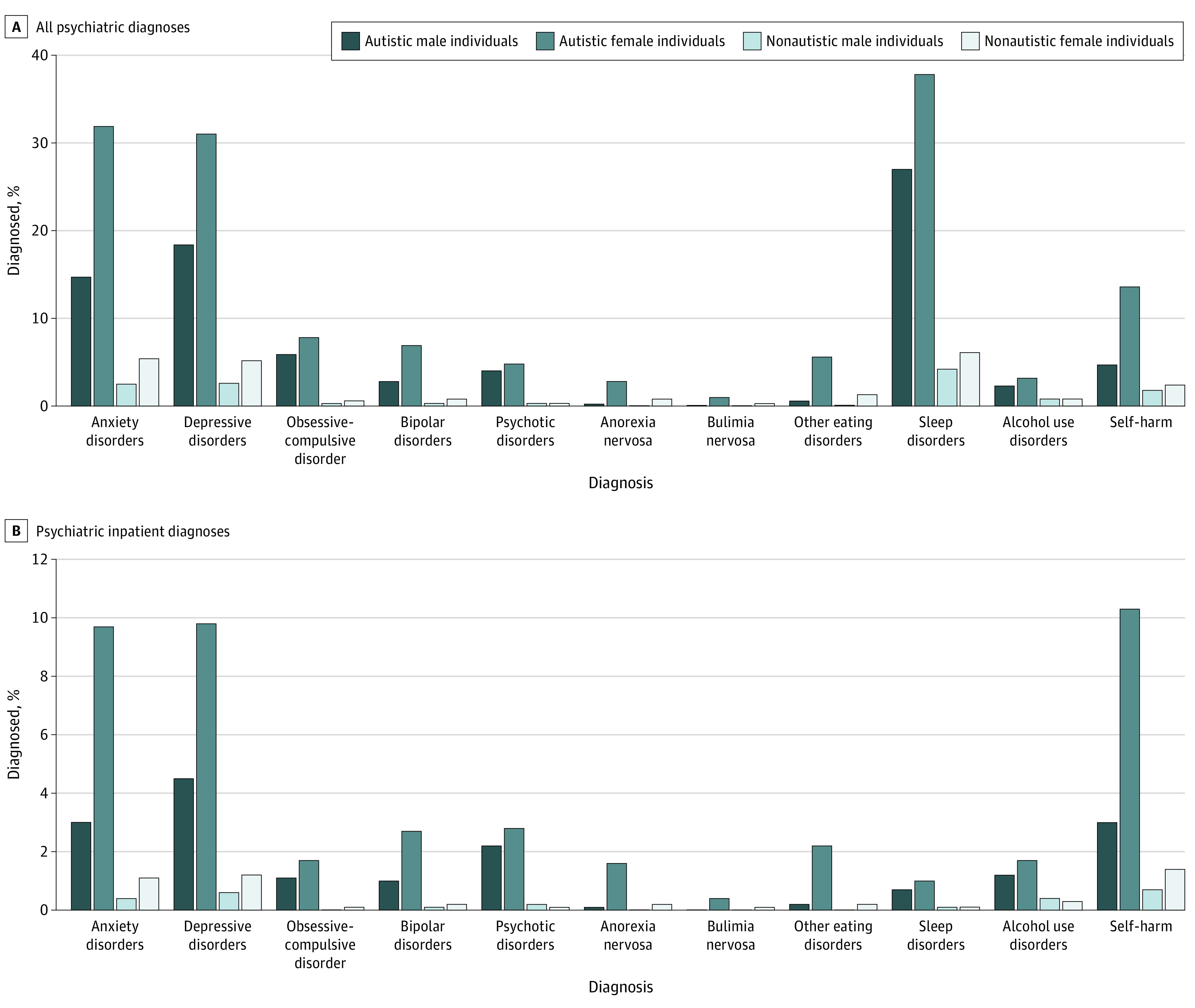
Percentages of Individual Psychiatric Diagnoses

Descriptive statistics for the study population are presented in Table 1. The cohort included 1 335 753 individuals (650 314 [48.7%] female). Detailed information on race and ethnicity was not available. Autism was clinically diagnosed in 20 841 individuals (1.6%; 7129 [34.2%] female) with a mean (SD) age of 16.1 (5.1) years (17.0 [4.8] years in female individuals and 15.7 [5.2] years in male individuals) for the first recorded autism diagnosis. Percentages of individuals receiving each psychiatric diagnosis are presented in eTable 4 in the Supplement and Figure 1.

**Table 1.** Demographic and Descriptive Characteristics of the Study Cohort

Characteristic	No. (%)					
	Autism diagnosis		Male		Female	
	No	Yes	Nonautistic	Autistic	Nonautistic	Autistic
No. (%)	1 314 912 (98.4)	20 841 (1.6)	671 727 (51.1)	13 712 (65.8)	643 185 (48.9)	7129 (34.2)
Birth year						
1985-1989	497 843 (37.9)	6064 (29.1)	254 984 (38.0)	3900 (28.4)	242 859 (37.8)	2164 (30.4)
1990-1993	450 091 (34.2)	7352 (35.3)	230 081 (34.3)	4754 (34.7)	220 010 (34.2)	2598 (36.4)
1994-1997	366 978 (27.9)	7425 (35.6)	186 662 (27.8)	5058 (36.9)	180 316 (28.0)	2367 (33.2)
Age at first recorded autism diagnosis, mean (SD), y	NA	16.11 (5.1)	NA	15.65 (5.2)	NA	16.99 (4.8)
ADHD diagnosis	47 763 (3.6)	9371 (45.0)	29 053 (4.3)	6178 (45.1)	18 710 (2.9)	3193 (44.8)
ID diagnosis	9545 (0.7)	3689 (17.7)	5342 (0.8)	2488 (18.1)	4203 (0.7)	1201 (16.8)
Psychiatric diagnosis^a^						
Any psychiatric disorder	143 963 (10.9)	10 582 (50.8)	57 896 (8.6)	6151 (44.9)	86 067 (13.4)	4431 (62.2)
Anxiety disorders	51 572 (3.9)	4294 (20.6)	16 847 (2.5)	2020 (14.7)	34 725 (5.4)	2274 (31.9)
Depressive disorders	50 734 (3.9)	4734 (22.7)	17 258 (2.6)	2523 (18.4)	33 476 (5.2)	2211 (31.0)
Obsessive-compulsive disorder	6352 (0.5)	1356 (6.5)	2341 (0.3)	803 (5.9)	4011 (0.6)	553 (7.8)
Bipolar disorder	7090 (0.5)	880 (4.2)	2042 (0.3)	388 (2.8)	5048 (0.8)	492 (6.9)
Psychotic disorders	3895 (0.3)	892 (4.3)	2225 (0.3)	551 (4.0)	1670 (0.3)	341 (4.8)
Anorexia nervosa	5119 (0.4)	234 (1.1)	229 (0.0)	32 (0.2)	4890 (0.8)	202 (2.8)
Bulimia nervosa	2166 (0.2)	76 (0.4)	49 (0.0)	7 (0.1)	2117 (0.3)	69 (1.0)
Other eating disorders	8675 (0.7)	481 (2.3)	435 (0.1)	84 (0.6)	8240 (1.3)	397 (5.6)
Sleep disorders	67 239 (5.1)	6400 (30.7)	27 948 (4.2)	3702 (27.0)	39 291 (6.1)	2698 (37.8)
Alcohol use disorders	10 317 (0.8)	537 (2.6)	5353 (0.8)	312 (2.3)	4964 (0.8)	225 (3.2)
Self-harm	27 581 (2.1)	1616 (7.8)	12 244 (1.8)	645 (4.70)	15 337 (2.4)	971 (13.6)

Abbreviations: ADHD, attention-deficit/hyperactivity disorder; ID, intellectual disability; NA, not applicable.

^a^ All psychiatric diagnoses received in inpatient or outpatient care.

### Sex Differences in Psychiatric Diagnoses

**Figure 2.**
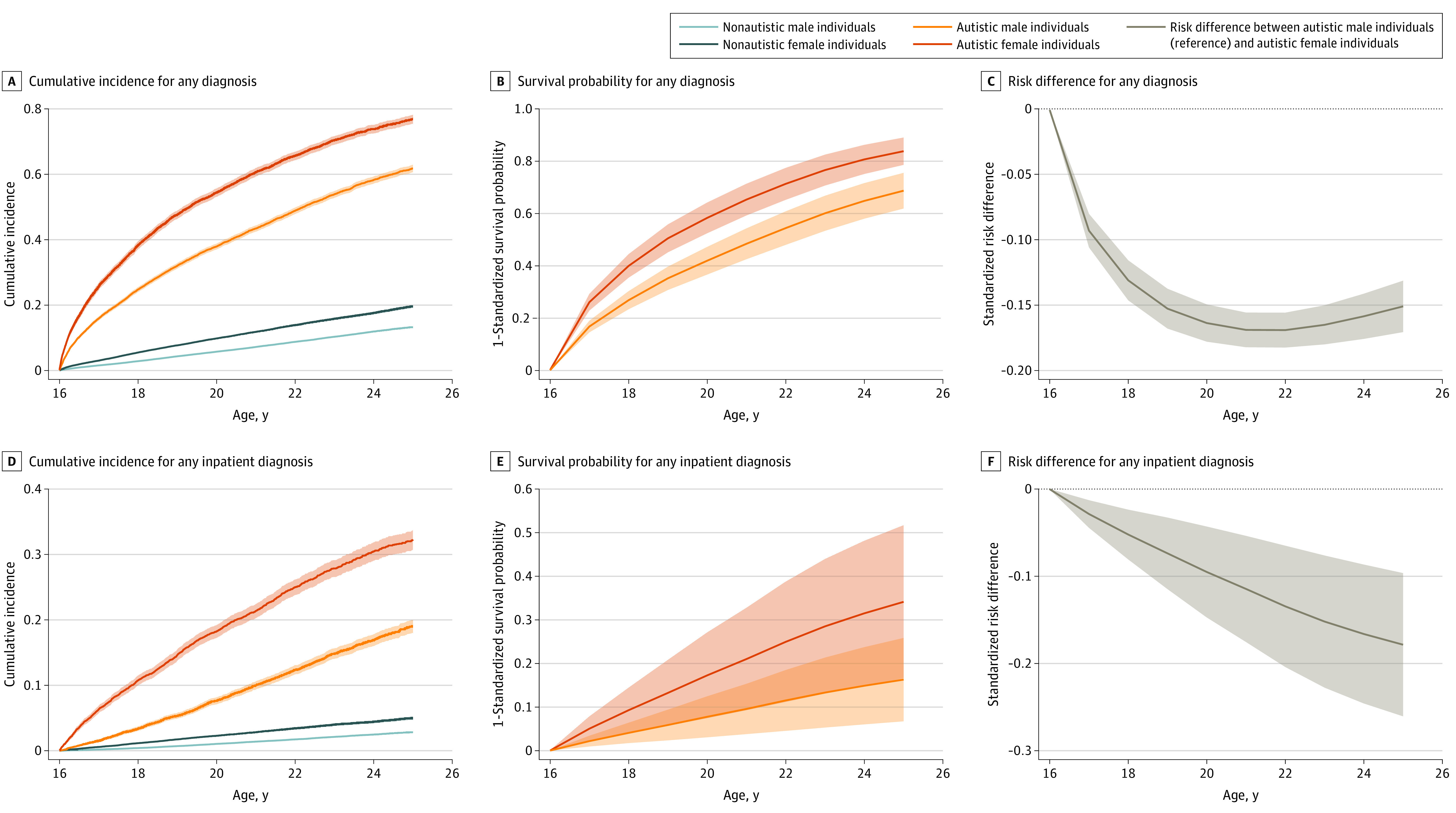
Cumulative Incidence, Birth Year–Standardized Survival Probability, and Risk Difference for Any Disorder A-C, Cumulative incidence, 1 − birth year–standardized survival probability, and risk difference for any inpatient or outpatient diagnosis, whereas D-F includes any inpatient diagnosis.

We observed sex differences in the cumulative incidence of psychiatric diagnoses between age 16 and 25 years among autistic individuals: 77 of 100 autistic female individuals, compared with 62 of 100 autistic male individuals, received at least 1 psychiatric diagnosis. Cumulative incidence was higher for autistic female individuals (0.016 [95% CI, 0.012-0.020]-0.52 [95% CI, 0.50-0.53]) than autistic male individuals (0.001 [95% CI, 0.000-0.002]-0.39 [95% CI, 0.38-0.40]) and nonautistic individuals across all individual disorders (eTable 5 in the Supplement). Comparing the standardized survival probability of autistic female and male individuals at age 25 years, we observed a statistically significant standardized risk difference for any diagnosis (−0.15; 95% CI, −0.17 to −0.13), indicating higher risk in female individuals (Figure 2). The same pattern was seen for sleep, depressive, and anxiety disorders (−0.28 [95% CI, −0.34 to −0.23] to −0.12 [95% CI, −0.14 to −0.11]; eFigure 2 in the Supplement). For most disorders risk differences between autistic female and male individuals were in the same direction yet larger than in nonautistic individuals (range of standardized risk differences at age 25 years: autistic individuals, −0.28 [95% CI, −0.34 to −0.23] to −0.007 [95% CI, −0.03 to 0.01]; nonautistic individuals, −0.10 [95% CI, −0.16 to −0.05] to 0.002 [95% CI, −0.03 to 0.03]; eTable 6 in the Supplement).

**Figure 3.**
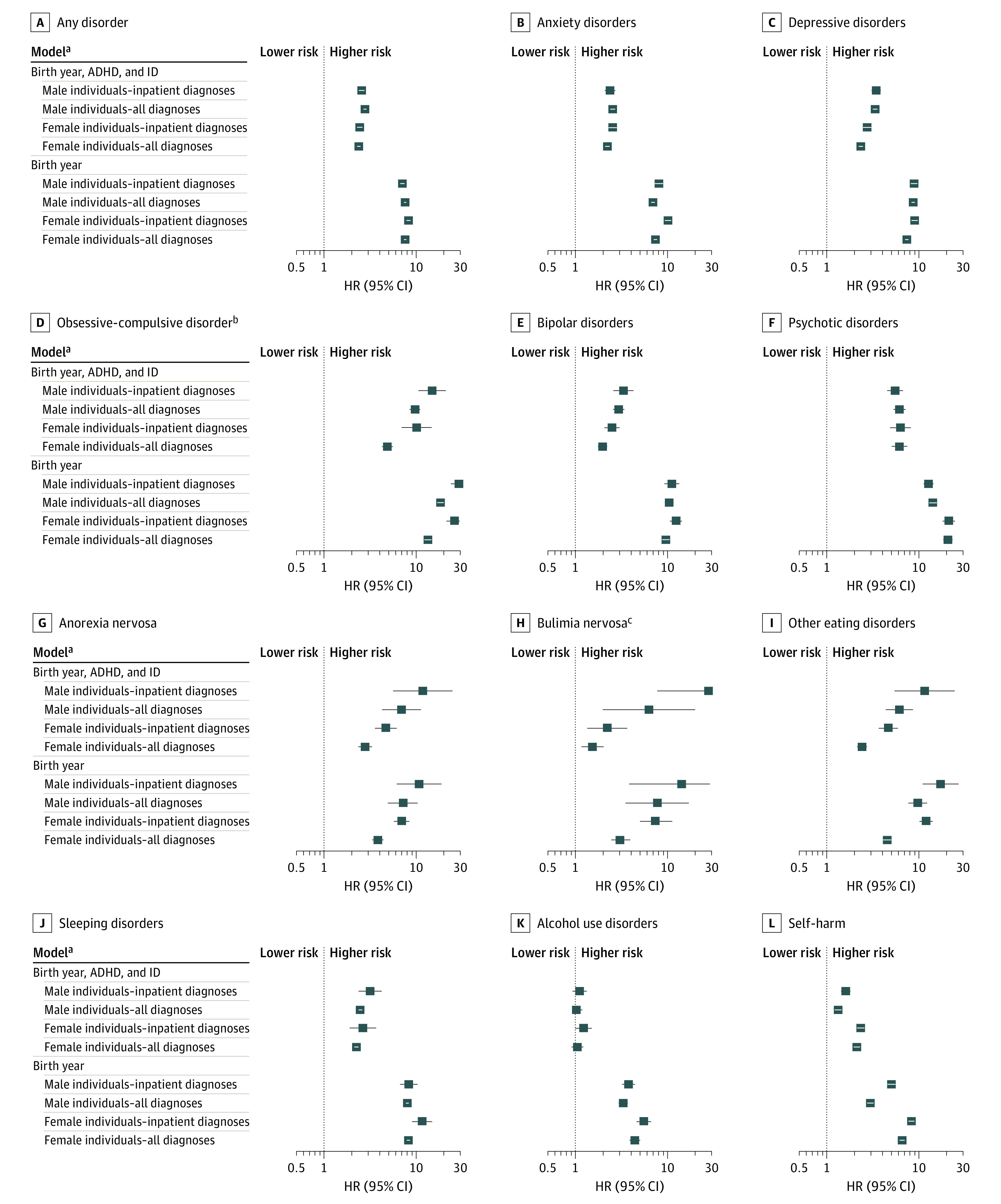
Risk of Psychiatric Diagnoses in Autistic Compared With Nonautistic Individuals Hazard ratios (HRs) comparing autistic and nonautistic individuals of the same sex are reported here. Confidence intervals exceed the plot limits and are cut at 30. See eTable 12 in the Supplement for more information. ADHD indicates attention-deficit/hyperactivity disorder; HR, hazard ratio; ID, intellectual disability. ^a^All diagnoses indicates the inclusion of both outpatient and inpatient diagnoses. Inpatient indicates that only inpatient diagnoses were considered. ^b^Model birth year, male individuals–inpatient diagnoses: upper CI = 35.87 and female individuals–inpatient diagnoses: upper CI = 32.16. ^c^Model birth year, male individuals–all diagnoses: upper CI = 54.49 and model birth year, ADHD, and ID, male individuals–all diagnoses: upper CI = 105.04.

Sex-specific birth year-adjusted HRs showed an elevated relative risk for all disorders for autistic female individuals (HR range [95% CI], 3.17 [2.50-4.04]-20.78 [18.48-23.37]) and male individuals (HR range [95% CI], 2.98 [2.75-3.23]-18.52 [17.07-20.08]) compared with same-sex individuals without an autism diagnosis (Figure 3, model birth year). When adjusting for ADHD and ID, most of the HRs for female and male individuals remained statistically significant, except for alcohol use disorders (Figure 3, model birth year, ADHD, and ID; eTable 7 in the Supplement). The results of the sensitivity analyses including only individuals diagnosed before age 16 years are shown in Table 2 and eFigure 3 in the Supplement. Despite attenuated HRs, autistic female individuals showed higher cumulative incidences for all disorders except alcohol use disorders compared with autistic male individuals. Results from analyses including only individuals with multiple autism diagnoses resembled the main analyses (eTable 8 in the Supplement).

**Table 2.** Sensitivity Analysis Including Only Autistic Individuals Diagnosed Before Age 16 Years, Cox Regression Estimates Stratified by Sex for All Psychiatric Diagnoses^a^

Psychiatric disorder	Male		Female		Male vs female	
	HR (95% CI)	*P* value	HR (95% CI)	*P* value	HR (95% CI)	*P* value
**Model adjusted for birth year**						
Any disorder	5.77 (5.51-6.04)	<.001	5.30 (4.98-5.65)	<.001	0.92 (0.85-0.99)	.03
Anxiety disorders	3.59 (3.27-3.95)	<.001	3.88 (3.52-4.28)	<.001	1.08 (0.94-1.23)	.27
Depressive disorders	4.36 (4.01-4.74)	<.001	3.97 (3.60-4.39)	<.001	0.91 (0.80-1.04)	.16
Obsessive-compulsive disorder	14.80 (13.05-16.79)	<.001	9.29 (7.73-11.17)	<.001	0.63 (0.50-0.78)	<.001
Bipolar disorders	7.82 (6.50-9.41)	<.001	7.32 (6.05-8.84)	<.001	0.94 (0.72-1.22)	.62
Psychotic disorders	7.02 (5.74-8.57)	<.001	12.86 (9.89-16.73)	<.001	1.83 (1.32-2.55)	<.001
Anorexia nervosa	3.44 (1.61-7.32)	.001	2.04 (1.47-2.83)	<.001	0.59 (0.26-1.35)	.22
Bulimia nervosa	2.82 (0.40-20.17)	.30	1.14 (0.54-2.39)	.74	0.40 (0.05-3.29)	.40
Other eating disorders	4.88 (3.12-7.65)	<.001	2.17 (1.70-2.78)	<.001	0.45 (0.27-0.74)	.002
Sleep disorders	7.12 (6.74-7.51)	<.001	6.72 (6.23-7.26)	<.001	0.95 (0.86-1.04)	.24
Alcohol use disorders	1.57 (1.21-2.05)	.001	1.40 (0.90-2.17)	.13	0.89 (0.53-1.49)	.65
Self-harm	1.86 (1.59-2.18)	<.001	4.04 (3.47-4.70)	<.001	2.17 (1.74-2.70)	<.001
**Model adjusted for birth year, ADHD, and ID**						
Any disorder	2.04 (1.93-2.16)	<.001	1.71 (1.58-1.84)	<.001	0.84 (0.76-0.92)	<.001
Anxiety disorders	1.25 (1.13-1.38)	<.001	1.18 (1.06-1.32)	.003	0.95 (0.81-1.10)	.47
Depressive disorders	1.56 (1.42-1.72)	<.001	1.28 (1.15-1.43)	<.001	0.82 (0.71-0.95)	.008
Obsessive-compulsive disorder	5.78 (4.87-6.85)	<.001	2.92 (2.36-3.63)	<.001	0.51 (0.38-0.67)	<.001
Bipolar disorders	2.18 (1.78-2.67)	<.001	1.69 (1.38-2.08)	<.001	0.77 (0.58-1.03)	.08
Psychotic disorders	2.05 (1.62-2.59)	<.001	2.43 (1.77-3.32)	<.001	1.18 (0.80-1.75)	.40
Anorexia nervosa	3.02 (1.28-7.13)	.01	1.46 (1.03-2.08)	.04	0.48 (0.19-1.22)	.12
Bulimia nervosa	1.90 (0.26-14.00)	.53	0.65 (0.31-1.40)	.28	0.34 (0.04-2.92)	.33
Other eating disorders	2.58 (1.53-4.33)	<.001	1.20 (0.92-1.56)	.18	0.47 (0.26-0.83)	.01
Sleep disorders	2.15 (2.02-2.29)	<.001	1.90 (1.73-2.08)	<.001	0.88 (0.79-0.99)	.03
Alcohol use disorders	0.54 (0.41-0.71)	<.001	0.39 (0.25-0.60)	<.001	0.72 (0.42-1.21)	.21
Self-harm	0.87 (0.74-1.03)	.11	1.36 (1.16-1.61)	<.001	1.56 (1.24-1.97)	<.001

Abbreviations: ADHD, attention-deficit/hyperactivity disorder; HR, hazard ratio; ID, intellectual disability.

^a^ N = 1 324 659; autistic individuals: n = 9747.

### Sex Differences in Psychiatric Hospitalizations

Percentages of psychiatric hospitalizations are presented in eTable 9 in the Supplement and Figure 1. By age 25 years, 32 of 100 autistic female individuals compared with 19 autistic male individuals, 5 nonautistic female individuals, and 3 nonautistic male individuals were hospitalized due to any psychiatric disorder. Cumulative incidence for inpatient diagnoses was higher in autistic female individuals for all individual disorders (cumulative incidence at age 25 years [95% CI], 0.01 [0.004-0.009]-0.16 [0.14-0.17]), compared with autistic male individuals and nonautistic individuals (eTable 10 in the Supplement). The standardized risk difference comparing autistic female and male individuals was statistically significant for any psychiatric disorder (−0.18; 95% CI, −0.26 to −0.10; Figure 2) but not for individual disorders (eFigure 4 in the Supplement). However, all risk differences showed a higher absolute risk for female individuals. Standardized risk differences were larger for autistic compared with nonautistic individuals (range of standardized risk differences at age 25 years: autistic individuals, −0.18 [95% CI, −0.26 to −0.10] to −0.006 [95% CI, −0.04 to 0.03]; nonautistic individuals, −0.03 [95% CI, −0.17 to 0.11] to 0.002 [95% CI, −0.04 to 0.04]; eTable 11 in the Supplement).

Sex-specific birth year–adjusted HRs indicated a higher risk of hospitalizations for autistic female and male individuals compared with same-sex individuals without autism for all inpatient diagnoses (female individuals: HR range [95% CI], 5.55 [4.63-6.66]-26.30 [21.50-32.16]; male individuals: HR range [95% CI], 3.79 [3.22-4.45]-29.36 [24.04-35.87]; Figure 3, model birth year). After adjusting for ADHD and ID, sex-specific HRs remained statistically significant for all disorders, except alcohol use disorder (Figure 3, model birth year, ADHD, and ID; eTable 12 in the Supplement).

## Discussion

To our knowledge, this is the largest study on sex differences in psychiatric disorders in autism to date, and the first study to comprehensively investigate psychiatric problems at different levels of psychiatric care in autistic young adults. Autistic young female individuals showed more mental health problems at multiple psychiatric care levels. Compared with autistic male individuals, autistic female individuals were at higher risk for any psychiatric disorder and specifically anxiety, depressive, and sleep disorders. They were also more likely to have been hospitalized for any psychiatric disorder compared with autistic male individuals and nonautistic individuals. Overall, sex differences observed between autistic female and male individuals resembled those in nonautistic individuals (namely higher incidence in female individuals for most disorders), but the differences in cumulative incidence were larger among autistic individuals. The findings of this large, population-based sample, including, to our knowledge, the highest number of autistic female individuals (n = 7129) studied so far, demonstrate a high level of psychiatric difficulties among young autistic female individuals, and thus clearly emphasize this group’s pressing mental health needs. Nevertheless, we need to consider psychiatric disorders in both sexes as psychiatric diagnoses and hospitalizations were more likely in autistic female and male individuals compared with nonautistic individuals of the same sex.

This investigation provides an important and novel contribution by exploring sex differences in psychiatric inpatient diagnoses. Our study crucially showed that psychiatric hospitalizations are relatively common in autistic young adults. By as young as age 25 years, 22.1% of autistic female individuals and 10.9% of autistic male individuals (compared with less than 4% among nonautistic individuals) had been hospitalized for psychiatric difficulties, which have the potential to worsen over the course of their lives, if not treated appropriately. These high hospitalization rates partly reflect the severity of the disorder, indicating severe mental health problems in autistic individuals, particularly autistic female individuals. However, person-level factors often interact with system-level factors, such as availability of and barriers to services.^31^ Facing these barriers might make autistic individuals more likely to delay and avoid health care, thereby exacerbating their mental health problems, potentially leading to acute psychiatric crises that require hospitalization.^31,32^ Which factors lead to hospitalization and whether autistic individuals benefit from inpatient treatment or whether the hospital environment negatively impacts their mental health should be addressed in future studies.

### Possible Underlying Mechanisms for the Observed Sex Differences

Different factors may exacerbate psychiatric disorders in autistic female individuals compared with male individuals. One theoretical approach is the multiple minority theory.^33^ Being autistic and nonmale can be viewed as a form of minority identity.^14^ Individuals with a minority identity tend to experience increased distress, which adversely impacts their mental health^33^ and could explain the results observed in this study. More proximal explanations, specifically related to the experience of being an autistic female individual, include female autism presentation^34^ (a qualitatively and/or quantitatively different expression of autistic symptoms and behaviors which might not be covered by current diagnostic criteria), compensatory behaviors and camouflaging, which may be more common in autistic female individuals,^35^ delays in diagnosis^36,37^ and access to support.^19^ These tend to be interrelated and impact mental health.^38,39^

Besides contributing to psychiatric disorders through delayed diagnosis and access to support,^19^ the diagnostic bias often observed in autism^36^ (ie, the earlier identification of autism in young male individuals), could have directly impacted our findings. Likelihood for an autism diagnosis is increased in autistic female individuals presenting with additional problems.^39,40^ It has therefore been suggested that diagnosed female individuals represent the extreme end of the autistic female population.^41^ Consequently, autistic female individuals without such comorbidities may be missed and not diagnosed. If additional difficulties in the form of co-occurring disorders are inherent in diagnosed autistic female individuals, this might have introduced bias toward an overestimation of psychiatric disorders in this study. However, the percentages of co-occurring ADHD and ID in our study appeared similar for both autistic female and male individuals, and our estimates are in line with community-based samples recruited from outside clinics.^4^

### Clinical Implications

Results from this study can inform clinical practice in 2 important ways. First, services for autistic adults are scarce^42^ and barriers to care are pervasive, subsequently causing gaps and delays in treatment.^18,43^ Expanding mental health services in the transitional period from childhood to adulthood, particularly for female individuals, to reduce disruption and discontinuation of essential services is a necessary first step to accommodate the needs of autistic young adults.

Second, it is essential to tailor services to autistic individuals’ needs. Autistic people, particularly female individuals, often report a lack of autism knowledge and understanding of co-occurring psychiatric disorders^19,38,43,44,45^ among medical professionals, sometimes resulting in misdiagnosis.^46,47^ Improving communication between autistic individuals and medical staff is key, as miscommunication tends to complicate identification and management of co-occurring disorders.^48^

### Strengths and Limitations

The main strength of this study is the large nationwide sample, including a high number of autistic female individuals, which enabled us to comprehensively investigate psychiatric disorders, including rarer disorders. Using both outpatient and inpatient diagnoses based on reliable register data allowed us to investigate psychiatric problems at different psychiatric care levels and to draw more generalizable and robust conclusions. Nevertheless, the study is not without limitations.

We cannot exclude the possibility that autistic individuals in our cohort were misdiagnosed with psychiatric disorders or that autism was undiagnosed in individuals without an autism diagnosis. Misdiagnosis of psychiatric disorders,^47^ and co-occurring disorders overshadowing autistic traits,^39^ is relatively common in autistic individuals who often report disagreeing with assigned diagnoses.^46^ Psychiatric diagnoses have yet to be validated in autistic individuals. The extent to which this, together with differences in validity between outpatient and inpatient diagnoses, might have influenced our findings remains uncertain.

Although adjusting our analyses for ADHD/ID attenuated our estimates, we did not further stratify our analyses. How complex phenotypes with additional neurodevelopmental difficulties influence mental health in autism should be explored in future research. Initially, we aimed to account for the age of first recorded autism diagnosis, which was shown to influence co-occurring disorders in childhood.^15^ Underdiagnosis, misdiagnosis, or late diagnosis of autistic women^36,49^ alongside delays in support access^50^ may further exacerbate their psychiatric difficulties.^19,37^ However, outpatient care was only covered from 2001, restricting follow-up time. Earlier autism diagnoses, especially among older individuals, might have been missed, as indicated by the relatively high observed mean age of diagnosis. Studying the association of late diagnosis with mental health could help inferring mechanisms contributing to increased psychiatric difficulties in autistic female individuals.

Because no gender variable is available in the registers, we relied on sex assigned at birth to differentiate between female and male individuals. Importantly, as effects of sex and gender are entangled, this does not imply that the observed differences are solely due to biological mechanisms.^10^ This is relevant because a comparably higher proportion of autistic than nonautistic individuals do not identify with their assigned sex at birth or a binary gender.^51^ Nonbinary and nonconforming gender identity are particularly prevalent among autistic individuals assigned female at birth,^51^ potentially contributing to the observed sex differences. Findings from a study^14^ on mental health in autistic men, women, and nonbinary/transgender individuals indicated higher rates of psychiatric disorders for the latter 2, highlighting the need to identify to which degree autistic nonbinary/transgender individuals face additional barriers, stigma, and exacerbated psychiatric difficulties. Studies on intersectionality, including gender identity as well as other factors such as race, ethnicity, and socioeconomic status, their interaction and subsequent effect on autistic individuals’ mental health constitute important avenues for future research.

## Conclusions

In this cohort study, between ages 16 and 25 years, autistic female individuals experienced increased psychiatric difficulties at different levels of psychiatric care, from outpatient diagnoses to hospitalization, compared with autistic male individuals and nonautistic individuals. Higher rates compared with autistic male individuals were found for most psychiatric disorders with sex differences larger than among nonautistic individuals. This study expands the growing body of literature on autistic female individuals’ experiences and consequently the recognition of differing needs in this understudied and underserved group.

## Notes

Supplement. eAppendix. The Swedish Registers eTable 1. Excluded chromosomal abnormalities eTable 2. Diagnostic and pharmaceutical classification of examined psychiatric disorders eTable 3. Diagnostic classification of covariates eTable 4. Absolute numbers and percentages of all psychiatric diagnoses eTable 5. Cumulative incidence at age 25 for all psychiatric diagnoses by group and sex eTable 6. Sex-specific birth year-standardized cumulative incidence and risk difference for all psychiatric diagnoses comparing autistic and non-autistic individuals based on matched data eTable 7. Stratified cox regression estimates stratified by sex for all psychiatric diagnoses eTable 8. Sensitivity analysis including only autistic individuals diagnosed on more than one occasion -stratified cox regression estimates stratified by sex for all psychiatric diagnoses eTable 9. Absolute numbers and percentages of psychiatric inpatient diagnoses eTable 10. Cumulative incidence at age 25 for psychiatric inpatient diagnoses by group and sex eTable 11. Sex-specific birth year-standardized cumulative incidence and risk difference for inpatient psychiatric diagnoses comparing autistic and non-autistic individuals based on matched data eTable 12. Stratified cox regression estimates stratified by sex for inpatient diagnoses eFigure 1. Flow Diagram Cohort Selection Process eFigure 2. Cumulative Incidence, birth-year-standardized survival probability and risk difference for all diagnoses eFigure 3. Cumulative Incidence, birth-year-standardized survival probability and risk difference for all diagnoses including only autistic individuals diagnosed before age 16 eFigure 4. Cumulative Incidence, birth-year-standardized survival probability and risk difference for inpatient diagnoses eReferences
