# Supplementary material for: Sex Differences in Mental Health Problems and Psychiatric Hospitalization in Autistic Young Adults
Source: JAMA Psychiatry. 2022 Oct 26;79(12):1188–98. doi: 10.1001/jamapsychiatry.2022.3475 (PMC9607919; doi:10.1001/jamapsychiatry.2022.3475)
Supplement: Supplement. — eAppendix. The Swedish Registers eTable 1. Excluded chromosomal abnormalities eTable 2. Diagnostic and pharmaceutical classification of examined psychiatric disorders eTable 3. Diagnostic classification of covariates eTable 4. Absolute numbers and percentages of all psychiatric diagnoses eTable 5. Cumulative incidence at age 25 for all psychiatric diagnoses by group and sex eTable 6. Sex-specific birth year-standardized cumulative incidence and risk difference for all psychiatric diagnoses comparing autistic and non-autistic individuals based on matched data eTable 7. Stratified cox regression estimates stratified by sex for all psychiatric diagnoses eTable 8. Sensitivity analysis including only autistic individuals diagnosed on more than one occasion -stratified cox regression estimates stratified by sex for all psychiatric diagnoses eTable 9. Absolute numbers and percentages of psychiatric inpatient diagnoses eTable 10. Cumulative incidence at age 25 for psychiatric inpatient diagnoses by group and sex eTable 11. Sex-specific birth year-standardized cumulative incidence and risk difference for inpatient psychiatric diagnoses comparing autistic and non-autistic individuals based on matched data eTable 12. Stratified cox regression estimates stratified by sex for inpatient diagnoses eFigure 1. Flow Diagram Cohort Selection Process eFigure 2. Cumulative Incidence, birth-year-standardized survival probability and risk difference for all diagnoses eFigure 3. Cumulative Incidence, birth-year-standardized survival probability and risk difference for all diagnoses including only autistic individuals diagnosed before age 16 eFigure 4. Cumulative Incidence, birth-year-standardized survival probability and risk difference for inpatient diagnoses eReferences [file jamapsychiatry-e223475-s001.pdf]

Supplemental Online Content

Martini MI, Kuja-Halkola R, Butwicka A, et al. Sex differences in mental health problems and psychiatric hospitalization in autistic young adults. *JAMA Psychiatry*. Published online October 26, 2022.
doi:10.1001/jamapsychiatry.2022.3475

eAppendix. The Swedish Registers

eTable 1. Excluded chromosomal abnormalities

eTable 2. Diagnostic and pharmaceutical classification of examined psychiatric disord**ers**

eTable 3. Diagnostic classification of covariates

eTable 4. Absolute numbers and percentages of all psychiatric diagnoses

eTable 5. Cumulative incidence at age 25 for all psychiatric diagnoses by group and sex

eTable 6. Sex-specific birth year-standardized cumulative incidence and risk difference for all psychiatric diagnoses comparing autistic and non-autistic individuals based on matched data

eTable 7. Stratified cox regression estimates stratified by sex for all psychiatric diagnoses

eTable 8. Sensitivity analysis including only autistic individuals diagnosed on more than one occasion - stratified cox regression estimates stratified by sex for all psychiatric diagnoses

eTable 9. Absolute numbers and percentages of psychiatric inpatient diagnoses

eTable 10. Cumulative incidence at age 25 for psychiatric inpatient diagnoses by group and sex

eTable 11. Sex-specific birth year-standardized cumulative incidence and risk difference for inpatient psychiatric diagnoses comparing autistic and non-autistic individuals based on matched data

eTable 12. Stratified cox regression estimates stratified by sex for inpatient diagnoses

eFigure 1. Flow Diagram Cohort Selection Process

eFigure 2. Cumulative Incidence, birth-year-standardized survival probability and risk difference for all diagnoses

eFigure 3. Cumulative Incidence, birth-year-standardized survival probability and risk difference for all diagnoses including only autistic individuals diagnosed before age 16

eFigure 4. Cumulative Incidence, birth-year-standardized survival probability and risk difference for inpatient diagnoses

eReferences

This supplemental material has been provided by the authors to give readers additional information about their work.

© 2022 Martini M et al. *JAMA Psychiatry.*

eAppendix. The Swedish Registers

The Medical Birth Register (MBR)[1] records all births in Sweden since 1973. We obtained information on death and migration through linking the MBR to the Cause of Death Register and the Total Population Register (TPR)[2]. The National Patient Register (NPR)[3] covers nationwide inpatient treatment since 1987, and specialist outpatient care starting in 2001. Diagnoses are coded according to the Swedish version of the ICD-9 (1973 -1996) and ICD-10 (since 1997). The Prescribed Drug Register (PDR) contains information on dispensations of prescribed drugs, classified according to the Anatomical Therapeutic Chemical (ATC) classification system, in Sweden since July 2005.

eTable 1. Excluded chromosomal abnormalities

Classification	Diagnostic code	Diagnosis
ICD-9	758	Chromosomal anomalies<br>- Down's syndrome<br>- Patau's syndrome<br>- Edward's syndrome<br>- Autosomal deletion syndromes<br>- Balanced autosomal translocation in normal individual<br>- Other conditions due to autosomal anomalies<br>- Gonadal dysgenesis<br>- Klinefelter syndrome<br>- Other conditions due to sex chromosome anomalies<br>- Conditions due to anomaly of unspecified chromosome
ICD-10	Q90-99	Chromosomal abnormalities, not elsewhere classified<br>- Down syndrome<br>- Edwards syndrome and Patau syndrome<br>- Other trisomies and partial trisomies of the autosomes, not elsewhere classified<br>- Monosomies and deletions from the autosomes, not elsewhere classified<br>- Balanced rearrangements and structural markers, not elsewhere classified<br>- Turner syndrome<br>- Other sex chromosome abnormalities, female phenotype, not elsewhere classified<br>- Other sex chromosome abnormalities, male phenotype, not elsewhere classified<br>- Other chromosome abnormalities, not elsewhere classified

© 2022 Martini M et al. *JAMA Psychiatry.*

eTable 2. Diagnostic and pharmaceutical classification of examined psychiatric disorders.

ICD-9: Swedish version of the International Classification of Diseases, 9th revision; ICD-10: International Classification of Diseases, 10th revision
ATC: Anatomical Therapeutic Chemical (ATC) Classification System

Type of mental health problem	Classification	Diagnostic/ ACT code	Diagnosis/Drug
Anxiety disorders	ICD-9	300A<br>300C<br>300X	Anxiety states<br>Phobic disorders<br>Unspecified nonpsychotic mental disorder
	ICD-10	F40<br>F41	Phobic anxiety disorders<br>Other anxiety disorders
Depressive disorders	ICD-9	296B<br>300E<br>311	Major depressive disorder single episode<br>Dysthymic disorder<br>Depressive disorder, not elsewhere classified
	ICD-10	F32<br>F33<br>F34.1<br>F34.8<br>F34.9<br>F38<br>F39	Depressive episode<br>Recurrent depressive disorder<br>Dysthymia<br>Other persistent mood (affective) disorders<br>Persistent mood (affective) disorder, unspecified<br>Other mood (affective) disorders<br>Unspecified mood (affective) disorder
Obsessive compulsive disorder	ICD-9	300D	Obsessive-compulsive disorders
	ICD-10	F42	Obsessive-compulsive disorder
Bipolar disorders	ICD-9	296A<br>296C<br>296D<br>296E<br>296W<br>296X	Bipolar disorder I, single manic episode<br>Bipolar I disorder, most recent episode manic<br>Bipolar I disorder, most recent episode depressed<br>Bipolar I disorder, most recent episode mixed<br>Other and unspecified bipolar disorders<br>Other and unspecified episodic mood disorders
	ICD-10	F30<br>F31<br>F34.0	Manic episode<br>Bipolar affective disorder<br>Cyclothymia
	ATC	N05AN01	Lithium
Psychotic disorders	ICD-9	295<br>297<br>298	Schizophrenic disorders<br>Delusional disorders<br>Other nonorganic psychoses
	ICD-10	F20-29	Schizophrenia, schizotypal and delusional diosrders
	ATC	N05AH02	Clozapine
Anorexia nervosa	ICD-9	307B	Anorexia nervosa
	ICD-10	F50.0<br>F50.1	Anorexia nervosa<br>Atypical anorexia nervosa
Bulimia nervosa	ICD-10	F50.2<br>F50.3	Bulimia nervosa<br>Atypical bulimia nervosa
Other eating disorders	ICD-10	F50.9	Eating disorder, unspecified
Sleep disorders	ICD-9	307E<br>780F	Specific disorders of sleep of nonorganic origin<br>Sleep disturbances
	ICD-10	F51<br>G47	Nonorganic sleep disorders<br>Sleep disorders
	ATC	N05CH01<br>N05CF01<br>N05CF02<br>N05CF03	Melatonin<br>Zopiclone<br>Zolpidem<br>Zaleplon
Alcohol use disorders	ICD-9	291<br>303X<br>305A	Alcohol-induced mental disorders<br>Other and unspecified alcohol dependence<br>Nondependent alcohol abuse
	ICD-10	F10.1-F10.9	Mental and behavioural disorders due to use of alcohol (excluding acute intoxication)
Self-harm	ICD-9	E950-E959<br>E980-E989	Suicide And Self-Inflicted Injury<br>Injury Undetermined Whether Accidentally Or Purposely Inflicted
	ICD-10	X60-X84<br>Y87.0<br>Y10-Y34<br>Y87.2	Intentional self-harm<br>Sequelae of intentional self-harm, assault and events of undetermined intent<br>Event of undetermined intent<br>Sequelae of events of undetermined intent

© 2022 Martini M et al. *JAMA Psychiatry.*

eTable 3. Diagnostic classification of covariates.

Covariate	Classficiation	Diagnostic code
Attention-deficit hyperactivity disorder (ADHD) diagnosis and medication	ICD-9	314 Hyperkinetic syndrome of childhood
	ICD-10	F90 Hyperkinteic disorders
	ATC	N06BA04 Methylphenidate<br>N06BA01 Amphetamine<br>N06BA02 Dexamphetamine<br>N06BA09 Atomoxetine<br>N06BA12 Lisdexamfetamine
Intellectual disability	ICD-9	317-319 Intellectual disabilities
	ICD-10	F70-F79 Mental retardation

© 2022 Martini M et al. *JAMA Psychiatry.*

eTable 4. Absolute numbers and percentages of all psychiatric diagnoses.

	Non-autistic overall[a]	Autism overall[b]	Non-autistic males	Autistic males	Non-autistic females	Autistic females
N (%)	1,314,912 (98.4)[c]	20,841 (1.6)[c]	671,727 (51.1)[d]	13,712 (65.8)[e]	643,185 (48.9)[d]	7,129 (34.2)[e]
**Psychiatric diagnosis (%)**						
Any psychiatric disorder	143,963 (10.9)	10,582 (50.8)	57,896 (8.6)	6,151 (44.9)	86,067 (13.4)	4,431 (62.2)
Anxiety disorders	51,572 (3.9)	4,294 (20.6)	16,847 (2.5)	2,020 (14.7)	34,725 (5.4)	2,274 (31.9)
Depressive disorders	50,734 (3.9)	4,734 (22.7)	17,258 (2.6)	2,523 (18.4)	33,476 (5.2)	2,211 (31.0)
Obsessive-compulsive disorder	6,352 (0.5)	1,356 (6.5)	2,341 (0.3)	803 (5.9)	4,011 (0.6)	553 (7.8)
Bipolar disorder	7,090 (0.5)	880 (4.2)	2,042 (0.3)	388 (2.8)	5,048 (0.8)	492 (6.9)
Psychotic disorders	3,895 (0.3)	892 (4.3)	2,225 (0.3)	551 (4.0)	1,670 (0.3)	341 (4.8)
Anorexia nervosa	5,119 (0.4)	234 (1.1)	229 (0.0)	32 (0.2)	4,890(0.8)	202 (2.8)
Bulimia nervosa	2,166 (0.2)	76 (0.4)	49 (0.0)	7 (0.1)	2,117 (0.3)	69 (1.0)
Other eating disorders	8,675 (0.7)	481 (2.3)	435 (0.1)	84 (0.6)	8,240 (1.3)	397 (5.6)
Sleep disorders	67,239 (5.1)	6,400 (30.7)	27,948 (4.2)	3,702 (27.0)	39,291 (6.1)	2,698 (37.8)
Alcohol use disorders	10,317 (0.8)	537 (2.6)	5,353 (0.8)	312 (2.3)	4,964 (0.8)	225 (3.2)
Self-harm	27,581 (2.1)	1,616 (7.8)	12,244 (1.8)	645 (4.7)	15,337 (2.4)	971 (13.6)
**Number of diagnoses (%)[f]**						
Mean number of diagnoses (SD)	0.18 (0.63)	1.03 (1.37)	0.13 (0.49)	0.81 (1.15)	0.24 (0.74)	1.46 (1.63)
0	1,170,949 (89.1)	10,259 (49.2)	613,831 (91.4)	7,561 (55.1)	557,118 (86.6)	2,698 (37.8)
1	87,913 (6.7)	4,879 (23.4)	39,478 (5.9)	3,238 (23.6)	48,435 (7.5)	1,641 (23.0)
2	30,914 (2.4)	2,769 (13.3)	11,159 (1.7)	1,632 (11.9)	19,755 (3.1)	1,137 (15.9)
3	14,840 (1.1)	1,562 (7.5)	4,777 (0.7)	789 (5.8)	10,063 (1.6)	773 (10.8)
4	6,603 (0.5)	775 (3.7)	1,759 (0.3)	316 (2.3)	4,844 (0.8)	459 (6.4)
5	2,503 (0.2)	390 (1.9)	560 (0.1)	131 (1.0)	1,943 (0.3)	259 (3.6)
6	870 (0.1)	129 (0.6)	137 (0.0)	36 (0.3)	733 (0.1)	93 (1.3)
>6	320 (0.0)	78 (0.4)	26 (0.0)	9 (0.1)	294 (0.0)	69 (1.0)
**Mean age of diagnosis (SD)[g]**						
Any psychiatric disorder	19.58 (2.49)	18.42 (2.25)	19.86 (2.49)	18.58 (2.31)	19.39 (2.48)	18.20 (2.14)
Anxiety disorders	19.93 (2.43)	19.33 (2.39)	20.28 (2.43)	19.60 (2.42)	19.75 (2.41)	19.08 (2.33)
Depressive disorders	19.60 (2.47)	19.02 (2.37)	19.95 (2.46)	19.22 (2.44)	19.41(2.45)	18.79 (2.27)
Obsessive-compulsive disorder	19.55 (2.51)	18.87 (2.37)	19.48 (2.49)	18.70 (2.27)	19.59 (2.52)	19.12 (2.49)
Bipolar disorder	20.55 (2.49)	19.59 (2.54)	20.73 (2.48)	19.87 (2.73)	20.48 (2.48)	19.37 (2.36)
Psychotic disorders	20.48 (2.30)	19.70 (2.36)	20.72 (2.28)	19.86 (2.40)	20.15 (2.28)	19.43 (2.29)
Anorexia nervosa	18.27 (2.14)	18.29 (2.26)	18.40 (2.16)	18.19 (2.02)	18.26 (2.14)	18.30 (2.30)
Bulimia nervosa	20.06 (2.30)	19.65 (2.29)	20.53 (2.61)	18.84 (2.61)	20.05 (2.29)	19.73 (2.26)
Other eating disorders	19.02 (2.36)	18.84 (2.42)	18.95 (2.51)	18.84 (2.41)	19.02 (2.36)	18.84 (2.43)
Sleep disorders	20.34 (2.51)	18.89 (2.40)	20.38 (2.55)	18.89 (2.45)	20.30 (2.49)	18.89 (2.34)
Alcohol use disorders	20.44 (2.28)	20.46 (2.19)	20.70 (2.31)	20.67 (2.18)	20.16 (2.20)	20.17 (2.18)
Self-harm	19.44 (2.32)	19.05 (2.26)	19.73 (2.34)	19.61 (2.28)	19.21 (2.27)	18.68 (2.17)

[a] Individuals who did not receive a lifetime diagnosis of autism. [b] Individuals who received a diagnosis of autism either preceding or following a psychiatric diagnosis. [c] Percentage based on the whole sample (N= 1 335 753). [d] Percentage based on the overall number of individuals in the general population ($N_{population}$ = 1 314 912). [e] Percentage based on the overall number of autistic individuals (N_{autism} = 20 841). [f] Number of psychiatric diagnoses received by one individual.

[g] Mean age at the first diagnosis for each psychiatric disorder.

© 2022 Martini M et al. *JAMA Psychiatry.*

eTable 5. Cumulative incidence at age 25 for all psychiatric diagnoses by group and sex.

	Non-autistic males	Autism males	Non-autistic females	Autistic females
**Psychiatric diagnosis**				
Any psychiatric disorder	0.1329 [0.1303; 0.1355]	0.6178 [0.6062; 0.6290]	0.1959 [0.1918; 0.1999]	0.7685 [0.7550; 0.7813]
Anxiety disorders	0.0412 [0.0396; 0.0427]	0.2499 [0.2392; 0.2605]	0.0851 [0.0822; 0.0881]	0.4660 [0.4495; 0.4820]
Depressive disorders	0.0404 [0.0388; 0.0419]	0.2958 [0.2847; 0.3069]	0.0771 [0.0744; 0.0798]	0.4367 [0.4209; 0.4521]
Obsessive-compulsive disorder	0.0053 [0.0047; 0.0058]	0.0870 [0.0806; 0.0933]	0.0095 [0.0085; 0.0105]	0.1195 [0.1090; 0.1298]
Bipolar disorder	0.0053 [0.0047; 0.0058]	0.0527 [0.0469; 0.0584]	0.0130 [0.0117; 0.0142]	0.1078 [0.0979; 0.1176]
Psychotic disorders	0.0056 [0.0050; 0.0062]	0.0721 [0.0656; 0.0785]	0.0044 [0.0037; 0.0051]	0.0754 [0.0670; 0.0837]
Anorexia nervosa	0.0004 [0.0003; 0.0006]	0.0031 [0.0020; 0.0043]	0.0097 [0.0088; 0.0106]	0.0390 [0.0332; 0.0447]
Bulimia nervosa	0.0002 [0.0001; 0.0003]	0.0008 [0.0001; 0.0015]	0.0051 [0.0044; 0.0058]	0.0159 [0.0119; 0.0200]
Other eating disorders	0.0010 [0.0007; 0.0012]	0.0094 [0.0072; 0.0116]	0.0184 [0.0171; 0.0197]	0.0827 [0.0740; 0.0914]
Sleep disorders	0.0704 [0.0684; 0.0724]	0.3917 [0.3803; 0.4028]	0.0999 [0.0967; 0.1032]	0.5183 [0.5025; 0.5337]
Alcohol use disorders	0.0128 [0.0119; 0.0137]	0.0454 [0.0400; 0.0507]	0.0125 [0.0113; 0.0136]	0.0553 [0.0476; 0.0629]
Self-harm	0.0262 [0.0250; 0.0274]	0.0801 [0.0736; 0.0866]	0.0338 [0.0321; 0.0356]	0.1927 [0.1805; 0.2046]

© 2022 Martini M et al. *JAMA Psychiatry.*

eTable 6. Sex-specific birth year-standardized cumulative incidence and risk difference for all psychiatric diagnoses comparing autistic and non-autistic individuals based on matched data.

	Standardized cumulative incidence		Standardized risk difference	Standardized cumulative incidence		Standardized risk difference
	**Autistic males**	**Autistic females**	**Autistic individuals**	**Non-autistic males**	**Non-autistic females**	**Non-autistic individuals**
N	13,712	7,129		137,120	71,290	
**Psychiatric diagnosis**						
Any psychiatric disorder	0.687 (0.619;0.755)	0.838 (0.786;0.890)	-0.151 (-0.171;-0.131)	0.181 (0.049;0.312)	0.283 (0.098;0.468)	-0.102 (-0.156;-0.048)
Anxiety disorders	0.293 (0.159;0.427)	0.576 (0.393;0.759)	-0.283 (-0.336;-0.230)	0.061 (-0.136;0.257)	0.144 (-0.288;0.577)	-0.084 (-0.320;0.152)
Depressive disorders	0.320 (0.190;0.450)	0.519 (0.350;0.687)	-0.199 (-0.242;-0.155)	0.052 (-0.132;0.236)	0.114 (-0.270;0.498)	-0.062 (-0.262;0.138)
Obsessive-compulsive disorder	0.103 (-0.037;0.243)	0.131 (-0.043;0.306)	-0.028 (-0.066;0.009)	0.006 (-0.130;0.143)	0.012 (-0.242;0.266)	-0.006 (-0.123;0.112)
Bipolar disorder	0.057 (-0.037;0.152)	0.144 (-0.080;0.367)	-0.087 (-0.191;0.018)	0.009 (-0.193;0.211)	0.020 (-0.417;0.457)	-0.011 (-0.245;0.224)
Psychotic disorders	0.068 (-0.031;0.167)	0.076 (-0.033;0.184)	-0.007 (-0.025;0.010)	0.006 (-0.114;0.127)	0.005 (-0.087;0.096)	0.002 (-0.028;0.031)
Anorexia nervosa	0.003 (-0.018;0.024)	0.039 (-0.207;0.285)	-0.035 (-0.261;0.190)	0.000 (-0.041;0.042)	0.011 (-0.948;0.971)	-0.011 (-0.929;0.907)
Bulimia nervosa	0.001 (-0.005;0.006)	0.014 (-0.099;0.126)	-0.013 (-0.120;0.094)	0.000 (-0.003;0.003)	0.006 (-0.184;0.197)	-0.006 (-0.194;0.181)
Other eating disorders	0.011 (-0.042;0.064)	0.09 (-0.320;0.501)	-0.079 (-0.437;0.279)	0.001 (-0.069;0.071)	0.024 (-1.468;1.517)	-0.023 (-1.446;1.399)
Sleep disorders	0.524 (0.399;0.648)	0.645 (0.516;0.775)	-0.121 (-0.137;-0.105)	0.170 (-0.138;0.477)	0.226 (-0.150;0.602)	-0.056 (-0.126;0.013)
Alcohol use disorders	0.041 (-0.041;0.123)	0.053 (-0.053;0.150)	-0.012 (-0.040;0.012)	0.014 (-0.135;0.162)	0.014 (-0.143;0.172)	-0.001 (-0.010;0.009)
Self-harm	0.066 (-0.012;0.143)	0.209 (-0.015;0.432)	-0.143 (-0.290;0.004)	0.023 (-0.083;0.130)	0.036 (-0.126;0.197)	-0.012 (-0.067;0.042)

© 2022 Martini M et al. *JAMA Psychiatry.*

eTable 7. Stratified cox regression estimates stratified by sex for all psychiatric diagnoses.

	HR males [95% CI]	p_{males}	HR females [95% CI]	$p_{females}$	$HR_{males}/HR_{females}$	$p_{HRmales/HRfemales}$
**Crude model**						
Any disorder	7.94 [7.72, 8.17]	<.001	7.74 [7.49, 8.00]	<.001	0.97 [0.93, 1.02]	.231
Anxiety disorders	7.36 [7.03, 7.72]	<.001	7.68 [7.36, 8.02]	<.001	1.04 [0.98, 1.11]	.192
Depressive disorders	9.10 [8.73, 9.50]	<.001	7.62 [7.29, 7.96]	<.001	0.84 [0.79, 0.89]	<.001
Obsessive-compulsive disorder	19.37 [17.87, 20.99]	<.001	13.91 [12.72, 15.21]	<.001	0.72 [0.64, 0.81]	<.001
Bipolar disorders	11.00 [9.86, 12.26]	<.001	10.06 [9.16, 11.04]	<.001	0.91 [0.79, 1.06]	.222
Psychotic disorders	14.51 [13.22, 15.93]	<.001	20.66 [18.39, 23.22]	<.001	1.42 [1.23, 1.65]	<.001
Anorexia nervosa	7.39 [5.10, 10.69]	<.001	3.92 [3.40, 4.51]	<.001	0.53 [0.36, 0.79]	.002
Bulimia nervosa	8.06 [3.66, 17.71]	<.001	3.20 [2.52, 4.07]	<.001	0.40 [0.17, 0.90]	.028
Other eating disorders	10.41 [8.24, 13.15]	<.001	4.71 [4.26, 5.21]	<.001	0.45 [0.35, 0.58]	<.001
Sleep disorders	8.63 [8.33, 8.95]	<.001	8.55 [8.20, 8.92]	<.001	0.99 [0.94, 1.05]	.741
Alcohol use disorders	3.37 [3.00, 3.77]	<.001	4.52 [3.95, 5.16]	<.001	1.34 [1.13, 1.60]	.001
Self-harm	2.97 [2.74, 3.21]	<.001	6.56 [6.14, 7.00]	<.001	2.21 [1.99, 2.45]	<.001
**Model adjusted for birth year**						
Any disorder	7.64 [7.43, 7.86]	<.001	7.65 [7.41, 7.90]	<.001	1.00 [0.96, 1.04]	.948
Anxiety disorders	6.98 [6.66, 7.32]	<.001	7.43 [7.11, 7.75]	<.001	1.06 [1.00, 1.13]	.057
Depressive disorders	8.71 [8.35, 9.09]	<.001	7.43 [7.11, 7.76]	<.001	0.85 [0.80, 0.91]	<.001
Obsessive-compulsive disorder	18.52 [17.07, 20.08]	<.001	13.5 [12.34, 14.76]	<.001	0.73 [0.65, 0.82]	<.001
Bipolar disorders	10.47 [9.39, 11.68]	<.001	9.64 [8.78, 10.59]	<.001	0.92 [0.80, 1.06]	.259
Psychotic disorders	14.30 [13.01, 15.7]	<.001	20.78 [18.48, 23.37]	<.001	1.45 [1.25, 1.69]	<.001
Anorexia nervosa	7.18 [4.95, 10.43]	<.001	3.86 [3.35, 4.44]	<.001	0.54 [0.36, 0.80]	.002
Bulimia nervosa	7.96 [3.58, 17.68]	<.001	3.17 [2.50, 4.04]	<.001	0.40 [0.17, 0.92]	.031
Other eating disorders	9.77 [7.72, 12.36]	<.001	4.55 [4.11, 5.03]	<.001	0.47 [0.36, 0.60]	<.001
Sleep disorders	8.07 [7.79, 8.36]	<.001	8.29 [7.96, 8.64]	<.001	1.03 [0.97, 1.08]	.330
Alcohol use disorders	3.31 [2.96, 3.72]	<.001	4.43 [3.88, 5.07]	<.001	1.34 [1.12, 1.59]	.001
Self-harm	2.98 [2.75, 3.23]	<.001	6.61 [6.19, 7.05]	<.001	2.21 [2.00, 2.46]	<.001
**Model adjusted for birth year, ADHD[a] and ID[b]**						
Any disorder	2.79 [2.69, 2.90]	<.001	2.39 [2.29, 2.50]	<.001	0.86 [0.81, 0.91]	<.001
Anxiety disorders	2.55 [2.40, 2.71]	<.001	2.23 [2.11, 2.36]	<.001	0.88 [0.81, 0.95]	.002
Depressive disorders	3.36 [3.17, 3.56]	<.001	2.35 [2.22, 2.48]	<.001	0.70 [0.64, 0.76]	<.001
Obsessive-compulsive disorder	9.75 [8.52, 11.16]	<.001	4.89 [4.28, 5.60]	<.001	0.50 [0.41, 0.61]	<.001
Bipolar disorders	2.96 [2.57, 3.41]	<.001	1.98 [1.76, 2.22]	<.001	0.67 [0.56, 0.80]	<.001
Psychotic disorders	6.19 [5.32, 7.19]	<.001	6.18 [5.08, 7.51]	<.001	1.00 [0.78, 1.28]	.990
Anorexia nervosa	6.97 [4.29, 11.34]	<.001	2.80 [2.36, 3.33]	<.001	0.40 [0.24, 0.67]	.001
Bulimia nervosa	6.49 [2.04, 20.62]	.002	1.55 [1.18, 2.05]	.002	0.24 [0.07, 0.78]	.018
Other eating disorders	6.16 [4.39, 8.65]	<.001	2.42 [2.14, 2.74]	<.001	0.39 [0.27, 0.56]	<.001
Sleep disorders	2.49 [2.38, 2.61]	<.001	2.25 [2.14, 2.38]	<.001	0.91 [0.84, 0.97]	.006
Alcohol use disorders	1.04 [0.92, 1.19]	.507	1.05 [0.91, 1.22]	.488	1.01 [0.83, 1.23]	.923
Self-harm	1.33 [1.22, 1.45]	<.001	2.12 [1.96, 2.30]	<.001	1.60 [1.42, 1.80]	<.001

[a] Attention-deficit/hyperactivity disorder
[b] Intellectual disability

© 2022 Martini M et al. *JAMA Psychiatry.*

eTable 8. Sensitivity analysis including only autistic individuals diagnosed on more than one occasion -stratified cox regression estimates stratified by sex for all psychiatric diagnoses.

N =1,330,647 (n_{autism} = 15,735)	HR males [95% CI]	p_{males}	HR females [95% CI]	$p_{females}$	$HR_{females}/HR_{males}$	$p_{HRfemales/HRmales}$
**Model adjusted for birth year**						
Any disorder	9.05 [8.78, 9.33]	<.001	8.88 [8.57, 9.20]	<.001	0.98 [0.94, 1.03]	.428
Anxiety disorders	7.91 [7.52, 8.32]	<.001	8.34 [7.95, 8.74]	<.001	1.05 [0.98, 1.13]	.137
Depressive disorders	10.03 [9.58, 10.50]	<.001	8.35 [7.96, 8.75]	<.001	0.83 [0.78, 0.89]	<.001
Obsessive-compulsive disorder	22.12 [20.32, 24.08]	<.001	15.36 [13.97, 16.9]	<.001	0.69 [0.61, 0.79]	<.001
Bipolar disorders	12.24 [10.90, 13.74]	<.001	11.12 [10.06, 12.28]	<.001	0.91 [0.78, 1.06]	.217
Psychotic disorders	16.37 [14.81, 18.09]	<.001	24.3 [21.50, 27.48]	<.001	1.48 [1.27, 1.74]	<.001
Anorexia nervosa	8.04 [5.38, 12.01]	<.001	4.46 [3.84, 5.17]	<.001	0.55 [0.36, 0.85]	.007
Bulimia nervosa	7.57 [2.99, 19.15]	<.001	3.58 [2.77, 4.63]	<.001	0.47 [0.18, 1.24]	.129
Other eating disorders	10.53 [8.14, 13.62]	<.001	5.05 [4.53, 5.63]	<.001	0.48 [0.36, 0.63]	<.001
Sleep disorders	9.47 [9.11, 9.84]	<.001	9.47 [9.05, 9.91]	<.001	1.00 [0.94, 1.06]	.997
Alcohol use disorders	3.53 [3.11, 4.01]	<.001	4.75 [4.11, 5.50]	<.001	1.35 [1.11, 1.63]	.003
Self-harm	3.34 [3.07, 3.64]	<.001	7.42 [6.91, 7.96]	<.001	2.22 [1.99, 2.48]	<.001
**Model adjusted for birth year, ADHD and ID**						
Any disorder	3.02 [2.90, 3.15]	<.001	2.56 [2.44, 2.68]	<.001	0.85 [0.79, 0.90]	<.001
Anxiety disorders	2.67 [2.50, 2.84]	<.001	2.34 [2.2, 2.49]	<.001	0.88 [0.80, 0.96]	.004
Depressive disorders	3.57 [3.35, 3.80]	<.001	2.46 [2.31, 2.61]	<.001	0.69 [0.63, 0.75]	<.001
Obsessive-compulsive disorder	10.99 [9.50, 12.71]	<.001	5.19 [4.51, 5.97]	<.001	0.47 [0.39, 0.58]	<.001
Bipolar disorders	3.20 [2.76, 3.71]	<.001	2.16 [1.92, 2.44]	<.001	0.68 [0.56, 0.82]	<.001
Psychotic disorders	6.42 [5.48, 7.53]	<.001	6.71 [5.48, 8.21]	<.001	1.04 [0.81, 1.35]	.738
Anorexia nervosa	8.08 [4.67, 13.98]	<.001	3.18 [2.64, 3.83]	<.001	0.39 [0.22, 0.70]	.002
Bulimia nervosa	5.28 [1.49, 18.69]	.010	1.7 [1.27, 2.28]	<.001	0.32 [0.09, 1.18]	.087
Other eating disorders	6.26 [4.31, 9.09]	<.001	2.58 [2.26, 2.95]	<.001	0.41 [0.28, 0.61]	<.001
Sleep disorders	2.68 [2.55, 2.81]	<.001	2.4 [2.26, 2.54]	<.001	0.9 [0.83, 0.97]	.005
Alcohol use disorders	1.04 [0.90, 1.19]	.628	1.08 [0.92, 1.27]	.356	1.04 [0.84, 1.29]	.701
Self-harm	1.42 [1.29, 1.56]	<.001	2.26 [2.07, 2.46]	<.001	1.59 [1.40, 1.81]	<.001

© 2022 Martini M et al. *JAMA Psychiatry.*

eTable 9. Absolute numbers and percentages of psychiatric inpatient diagnoses.

	Non-autistic overall[a]	Autism overall[b]	Non-autistic males	Autistic males	Non-autistic females	Autistic females
N (%)	1,314,912 (98.4)[c]	20,841 (1.6)[c]	671,727 (51.1)[d]	13,712 (65.8)[e]	643,185 (48.9)[d]	7,129 (34.2)[e]
**Psychiatric diagnosis (%)**						
Any psychiatric disorder	33,436 (2.5)	3,063 (14.7)	12,389 (1.8)	1,488 (10.9)	21,047 (3.3)	1,575 (22.1)
Anxiety disorders	9,652 (0.7)	1,105 (5.3)	2,863 (0.4)	416 (3.0)	6,789 (1.1)	689 (9.7)
Depressive disorders	11,846 (0.9)	1,317 (6.3)	3,952 (0.6)	619 (4.5)	7,894 (1.2)	698 (9.8)
Obsessive-compulsive disorder	733 (0.1)	268 (1.3)	279 (0.0)	147 (1.1)	454 (0.1)	121 (1.7)
Bipolar disorder	2,173 (0.2)	324 (1.6)	662 (0.1)	132 (1.0)	1,511 (0.2)	192 (2.7)
Psychotic disorders	2,326 (0.2)	502 (2.4)	1,378 (0.2)	305 (2.2)	948 (0.1)	197 (2.8)
Anorexia nervosa	1,595 (0.1)	126 (0.6)	74 (0.0)	15 (0.1)	1,521 (0.2)	111 (1.6)
Bulimia nervosa	339 (0.0)	28 (0.1)	12 (0.0)	<5 (0.0)[f]	327 (0.1)	25 (0.4)
Other eating disorders	1,319 (0.1)	181 (0.9)	77 (0.0)	25 (0.2)	1,242 (0.2)	156 (2.2)
Sleep disorders	1,164 (0.1)	162 (0.8)	615 (0.1)	94 (0.7)	549 (0.1)	68 (1.0)
Alcohol use disorders	4,553 (0.3)	282 (1.4)	2,379 (0.4)	159 (1.2)	2,174 (0.3)	123 (1.7)
Self-harm	13,734 (1.0)	1,145 (5.5)	4,608 (0.7)	409 (3.0)	9,126 (1.4)	736 (10.3)
**Number of diagnoses (%)[g]**						
Mean number of diagnoses	0.04 (0.27)	0.26 (0.75)	0.03 (0.21)	0.17 (0.56)	0.05 (0.32)	0.44 (0.99)
0	1,281,476 (97.5)	17,778 (85.3)	659,338 (98.2)	12,224 (89.1)	622,138 (96.7)	5,554 (77.9)
1	22,625 (1.7)	1,653 (7.9)	9,082 (1.4)	930 (6.8)	13,543 (2.1)	723 (10.1)
2	7,129 (0.5)	798 (3.8)	2,369 (0.4)	360 (2.6)	4,760 (0.7)	438 (6.1)
3	2,571 (0.2)	369 (1.8)	721 (0.1)	135 (1.0)	1,850 (0.3)	234 (3.3)
4	799 (0.1)	169 (0.8)	177 (0.0)	50 (0.4)	622 (0.1)	119 (1.7)
5	245 (0.0)	46 (0.2)	33 (0.0)	9 (0.1)	212 (0.0)	37 (0.5)
6	54 (0.0)	20 (0.1)	6 (0.0)	<5 (0.0)[f]	48 (0.0)	16 (0.2)
> 6	13 (0.0)	8 (0.0)	<5 (0.0)[f]	0 (0.0)	12 (0.0)	8 (0.1)
**Mean age of diagnosis (SD)[h]**						
Any psychiatric disorder	19.75 (2.40)	19.28 (2.35)	20.21 (2.36)	19.75 (2.35)	19.47 (2.37)	18.83 (2.26)
Anxiety disorders	20.30 (2.41)	19.86 (2.41)	20.82 (2.33)	20.34 (2.37)	20.08 (2.40)	19.57 (2.40)
Depressive disorders	20.04 (2.38)	19.58 (2.42)	20.47 (2.31)	19.94 (2.41)	19.82 (2.38)	19.25 (2.40)
Obsessive-compulsive disorder	20.12 (2.46)	19.67 (2.42)	20.22 (2.41)	19.52 (2.28)	20.06 (2.49)	19.85 (2.59)
Bipolar disorder	20.73 (2.36)	20.01 (2.37)	21.00 (2.34)	20.46 (2.58)	20.61 (2.36)	19.70 (2.17)
Psychotic disorders	20.69 (2.27)	20.13 (2.38)	20.86 (2.24)	20.39 (2.32)	20.43 (2.29)	19.72 (2.42)
Anorexia nervosa	18.61 (2.19)	18.34 (2.17)	18.70 (2.04)	18.64 (2.38)	18.61 (2.19)	18.30 (2.14)
Bulimia nervosa	20.19 (2.27)	19.70 (2.46)	19.65 (2.40)	17.52 (0.87)	20.21 (2.27)	19.96 (2.46)
Other eating disorders	19.50 (2.34)	19.31 (2.31)	19.67 (2.59)	19.61 (2.01)	19.49 (2.33)	19.26 (2.36)
Sleep disorders	20.02 (2.57)	19.55 (2.52)	20.19 (2.53)	19.94 (2.51)	19.82 (2.60)	19.03 (2.46)
Alcohol use disorders	20.33 (2.31)	20.47 (2.26)	20.60 (2.34)	20.73 (2.28)	20.04 (2.24)	20.14 (2.19)
Self-harm	19.49 (2.32)	19.22 (2.25)	19.99 (2.33)	19.83 (2.28)	19.24 (2.27)	18.89 (2.17)

[a] Individuals who did not receive a lifetime diagnosis of autism. [b] Individuals who received a diagnosis of autism either preceding or following a psychiatric diagnosis. [c] Percentage based on the whole sample (N= 1 335 753). [d] Percentage based on the overall number of individuals in the general population ($N_{population}$ = 1 314 912). [e] Percentage based on overall number of autistic individuals (N_{autism} = 20 841). [f] To maintain the anonymity of our study cohort, frequencies lower than 5 are not presented. [g] Number of psychiatric diagnoses received by one individual. [h] Mean age at the first diagnosis for each psychiatric disorder.

© 2022 Martini M et al. *JAMA Psychiatry.*

eTable 10. Cumulative incidence at age 25 for psychiatric inpatient diagnoses by group and sex.

	Non-autistic males	Autistic males	Non-autistic females	Autistic females
**Psychiatric diagnosis**				
Any psychiatric disorder	0.0285 [0.0272; 0.0298]	0.1900 [0.1802; 0.1997]	0.0496 [0.0474; 0.0519]	0.3227 [0.3076; 0.3375]
Anxiety disorders	0.0077 [0.0070; 0.0084]	0.0585 [0.0525; 0.0645]	0.0174 [0.016; 0.0188]	0.1567 [0.1446; 0.1685]
Depressive disorders	0.0089 [0.0082; 0.0097]	0.0818 [0.0750; 0.0886]	0.0187 [0.0173; 0.0202]	0.1527 [0.1410; 0.1643]
Obsessive-compulsive disorder	0.0006 [0.0004; 0.0008]	0.0179 [0.0148; 0.0210]	0.0012 [0.0008; 0.0016]	0.0298 [0.0239; 0.0356]
Bipolar disorder	0.0018 [0.0014; 0.0021]	0.0193 [0.0157; 0.0229]	0.0043 [0.0036; 0.0051]	0.0436 [0.0371; 0.0502]
Psychotic disorders	0.0033 [0.0028; 0.0038]	0.0430 [0.0378; 0.0482]	0.0026 [0.0021; 0.0032]	0.0464 [0.0395; 0.0534]
Anorexia nervosa	0.0002 [0.0001; 0.0003]	0.0016 [0.0007; 0.0025]	0.0030 [0.0024; 0.0035]	0.0210 [0.0168; 0.0251]
Bulimia nervosa	0.0000 [0.0000; 0.0000]	0.0003 [0.0000; 0.0005]	0.0008 [0.0005; 0.0012]	0.0062 [0.0035; 0.0088]
Other eating disorders	0.0003 [0.0001; 0.0004]	0.0030 [0.0017; 0.0042]	0.0028 [0.0023; 0.0033]	0.0338 [0.0281; 0.0394]
Sleep disorders	0.0014 [0.0011; 0.0017]	0.0126 [0.0098; 0.0154]	0.0011 [0.0008; 0.0015]	0.0147 [0.0108; 0.0186]
Alcohol use disorders	0.0055 [0.0049; 0.0061]	0.0237 [0.0198; 0.0277]	0.0056 [0.0048; 0.0063]	0.0302 [0.0245; 0.0358]
Self-harm	0.0100 [0.0092; 0.0107]	0.0525 [0.0470; 0.0579]	0.0201 [0.0187; 0.0215]	0.1499 [0.1389; 0.1608]

© 2022 Martini M et al. *JAMA Psychiatry.*

eTable 11. Sex-specific birth year-standardized cumulative incidence and risk difference for inpatient psychiatric diagnoses comparing autistic and non-autistic individuals based on matched data.

	Standardized cumulative incidence	Standardized cumulative incidence	Standardized risk difference	Standardized cumulative incidence	Standardized cumulative incidence	Standardized risk difference
	**Autistic males**	**Autistic females**	**Autistic individuals**	**Non-autistic males**	**Non-autistic females**	**Non-autistic individuals**
N	13,712	7,129		137,120	71,290	
**Psychiatric diagnosis**						
Any psychiatric disorder	0.163 (0.067;0.258)	0.341 (0.166;0.517)	-0.178 (-0.261;-0.096)	0.028 (-0.105;0.160)	0.058 (-0.216;0.332)	-0.031 (-0.172;0.111)
Anxiety disorders	0.056 (-0.037;0.148)	0.187 (-0.097;0.471)	-0.132 (-0.324;0.061)	0.008 (-0.101;0.117)	0.027 (-0.326;0.380)	-0.019 (-0.262;0.225)
Depressive disorders	0.071 (-0.015;0.157)	0.162 (-0.023;0.347)	-0.091 (-0.191;0.009)	0.010 (-0.128;0.149)	0.025 (-0.297;0.346)	-0.014 (-0.197;0.169)
Obsessive-compulsive disorder	0.020 (-0.051;0.091)	0.034 (-0.084;0.152)	-0.014 (-0.062;0.034)	0.001 (-0.052;0.053)	0.001 (-0.067;0.069)	0.000 (-0.015;0.015)
Bipolar disorder	0.015 (-0.033;0.063)	0.046 (-0.099;0.190)	-0.031 (-0.127;0.066)	0.002 (-0.083;0.087)	0.006 (-0.240;0.252)	-0.004 (-0.165;0.157)
Psychotic disorders	0.033 (-0.027;0.094)	0.042 (-0.033;0.117)	-0.009 (-0.027;0.01)	0.005 (-0.111;0.120)	0.003 (-0.073;0.079)	0.002 (-0.038;0.041)
Anorexia nervosa[a]	0.001 (-0.005;0.007)	0.020 (-0.056;0.095)	-0.018 (-0.088;0.051)	0.000 (-0.007;0.007)	0.003 (-0.148;0.155)	-0.003 (-0.148;0.142)
Bulimia nervosa[b]	0.000 (-0.001;0.002)	0.006 (-0.030;0.042)	-0.006 (-0.04;0.029)			
Other eating disorders[a]	0.003 (-0.009;0.016)	0.037 (-0.093;0.166)	-0.033 (-0.15;0.084)	0.000 (-0.006;0.006)	0.003 (-0.098;0.104)	-0.003 (-0.098;0.092)
Sleep disorders	0.014 (-0.039;0.068)	0.025 (-0.067;0.117)	-0.011 (-0.05;0.029)	0.002 (-0.070;0.073)	0.001 (-0.049;0.052)	0.001 (-0.020;0.021)
Alcohol use disorders	0.020 (-0.035;0.074)	0.029 (-0.049;0.107)	-0.009 (-0.034;0.016)	0.006 (-0.092;0.104)	0.006 (-0.106;0.119)	-0.001 (-0.015;0.013)
Self-harm	0.042 (-0.020;0.103)	0.151 (-0.058;0.360)	-0.109 (-0.257;0.038)	0.008 (-0.081;0.098)	0.020 (-0.189;0.228)	-0.011 (-0.131;0.108)

[a] As there were no non-autistic males diagnosed for certain birth years we had to combine birth years into categories (1985-1989,1990-1993,1994-1997) for these disorders. We applied the same categories to autistic individuals to make the results comparable.

[b] No estimates could be provided for bulimia, as the number of diagnosed males was too low.

© 2022 Martini M et al. *JAMA Psychiatry.*

eTable 12. Stratified cox regression estimates stratified by sex for inpatient diagnoses.

	HR males [95% CI]	p_{males}	HR females [95% CI]	$p_{females}$	$HR_{males}/HR_{females}$	$p_{HRmales/HRfemales}$
**Crude model**						
Any disorder	7.24 [6.85, 7.64]	<.001	8.35 [7.93, 8.80]	<.001	1.15 [1.07, 1.24]	<.001
Anxiety disorders	8.48 [7.65, 9.41]	<.001	10.54 [9.74, 11.40]	<.001	1.24 [1.09, 1.41]	.001
Depressive disorders	9.13 [8.38, 9.94]	<.001	9.14 [8.45, 9.88]	<.001	1.00 [0.89, 1.12]	.984
Obsessive-compulsive disorder	29.62 [24.27, 36.15]	<.001	26.39 [21.61, 32.24]	<.001	0.89 [0.67, 1.18]	.423
Bipolar disorders	11.54 [9.57, 13.90]	<.001	12.83 [11.04, 14.91]	<.001	1.11 [0.88, 1.41]	.384
Psychotic disorders	12.90 [11.39, 14.6]	<.001	20.92 [17.94, 24.40]	<.001	1.62 [1.33, 1.98]	<.001
Anorexia nervosa	10.82 [6.21, 18.86]	<.001	6.95 [5.73, 8.43]	<.001	0.64 [0.36, 1.16]	.140
Bulimia nervosa	13.59 [3.88, 47.53]	<.001	7.51 [5.00, 11.29]	<.001	0.55 [0.15, 2.06]	.378
Other eating disorders	17.90 [11.39, 28.13]	<.001	12.25 [10.37, 14.47]	<.001	0.68 [0.42, 1.11]	.123
Sleep disorders	8.60 [6.91, 10.70]	<.001	12.10 [9.41, 15.57]	<.001	1.41 [1.01, 1.97]	.044
Alcohol use disorders	3.83 [3.26, 4.49]	<.001	5.58 [4.66, 6.69]	<.001	1.46 [1.15, 1.86]	.002
Self-harm	5.03 [4.54, 5.56]	<.001	8.21 [7.61, 8.85]	<.001	1.63 [1.44, 1.85]	<.001
**Model adjusted for birth year**						
Any disorder	7.13 [6.75, 7.53]	<.001	8.31 [7.89, 8.75]	<.001	1.17 [1.08, 1.26]	<.001
Anxiety disorders	8.10 [7.30, 8.99]	<.001	10.17 [9.40, 11.01]	<.001	1.26 [1.10, 1.43]	.001
Depressive disorders	8.91 [8.18, 9.70]	<.001	9.04 [8.36, 9.77]	<.001	1.01 [0.90, 1.14]	.805
Obsessive-compulsive disorder	29.36 [24.04, 35.87]	<.001	26.30 [21.50, 32.16]	<.001	0.90 [0.67, 1.19]	.446
Bipolar disorders	11.20 [9.28, 13.52]	<.001	12.41 [10.67, 14.43]	<.001	1.11 [0.87, 1.41]	.405
Psychotic disorders	12.78 [11.28, 14.48]	<.001	21.23 [18.18, 24.78]	<.001	1.66 [1.36, 2.03]	<.001
Anorexia nervosa	10.83 [6.18, 18.99]	<.001	7.00 [5.77, 8.50]	<.001	0.65 [0.36, 1.17]	.150
Bulimia nervosa	14.63 [3.93, 54.49]	<.001	7.66 [5.10, 11.50]	<.001	0.52 [0.13, 2.07]	.357
Other eating disorders	17.28 [11.02, 27.12]	<.001	12.03 [10.18, 14.21]	<.001	0.70 [0.43, 1.12]	.139
Sleep disorders	8.38 [6.72, 10.44]	<.001	11.69 [9.08, 15.04]	<.001	1.40 [1.00, 1.95]	.051
Alcohol use disorders	3.79 [3.22, 4.45]	<.001	5.55 [4.63, 6.66]	<.001	1.47 [1.15, 1.87]	.002
Self-harm	5.05 [4.57, 5.59]	<.001	8.33 [7.72, 8.99]	<.001	1.65 [1.45, 1.87]	<.001
**Model adjusted for birth year, ADHD and ID**						
Any disorder	2.57 [2.40, 2.76]	<.001	2.45 [2.29, 2.62]	<.001	0.95 [0.86, 1.05]	.325
Anxiety disorders	2.38 [2.09, 2.72]	<.001	2.55 [2.30, 2.82]	<.001	1.07 [0.91, 1.26]	.430
Depressive disorders	3.45 [3.07, 3.88]	<.001	2.75 [2.48, 3.05]	<.001	0.80 [0.68, 0.93]	.004
Obsessive-compulsive disorder	15.02 [10.65, 21.16]	<.001	10.19 [7.00, 14.84]	<.001	0.68 [0.41, 1.13]	.136
Bipolar disorders	3.34 [2.59, 4.29]	<.001	2.51 [2.07, 3.03]	<.001	0.75 [0.55, 1.03]	.075
Psychotic disorders	5.54 [4.55, 6.75]	<.001	6.34 [4.88, 8.22]	<.001	1.14 [0.83, 1.58]	.421
Anorexia nervosa	11.88 [5.65, 24.98]	<.001	4.71 [3.59, 6.18]	<.001	0.40 [0.18, 0.87]	.022
Bulimia nervosa	28.78 [7.89, 105.04]	<.001	2.26 [1.37, 3.73]	.001	0.08 [0.02, 0.31]	<.001
Other eating disorders	11.61 [5.46, 24.66]	<.001	4.67 [3.67, 5.94]	<.001	0.40 [0.18, 0.89]	.024
Sleep disorders	3.18 [2.38, 4.25]	<.001	2.65 [1.90, 3.70]	<.001	0.83 [0.54, 1.30]	.421
Alcohol use disorders	1.11 [0.93, 1.33]	.240	1.23 [1.00, 1.51]	.050	1.10 [0.84, 1.45]	.481
Self-harm	1.61 [1.43, 1.81]	<.001	2.34 [2.13, 2.57]	<.001	1.46 [1.25, 1.69]	<.001

© 2022 Martini M et al. *JAMA Psychiatry.*

eFigure 1. Flow Diagram Cohort Selection Process.

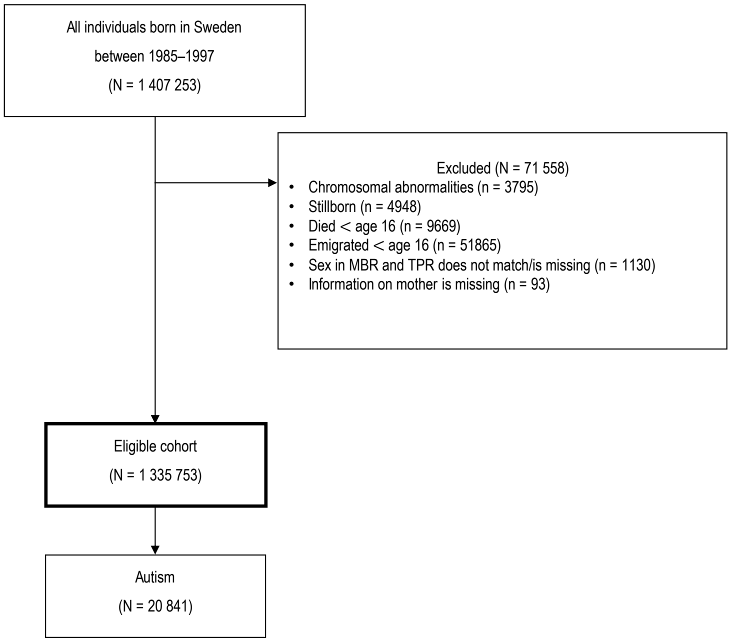

© 2022 Martini M et al. *JAMA Psychiatry.*

eFigure 2. Cumulative Incidence, birth-year-standardized survival probability and risk difference for all diagnoses.

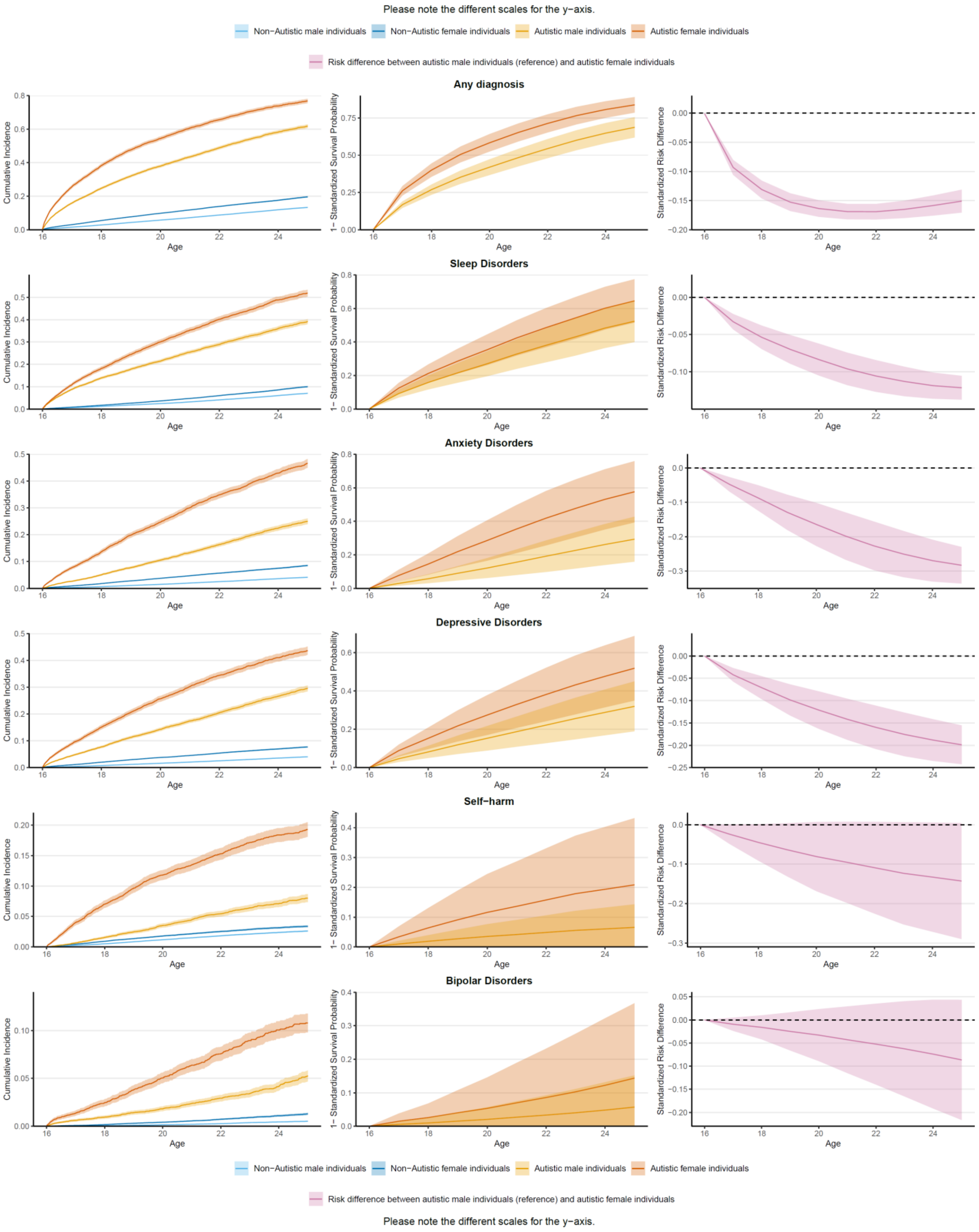

© 2022 Martini M et al. *JAMA Psychiatry.*

eFigure 2 continued.

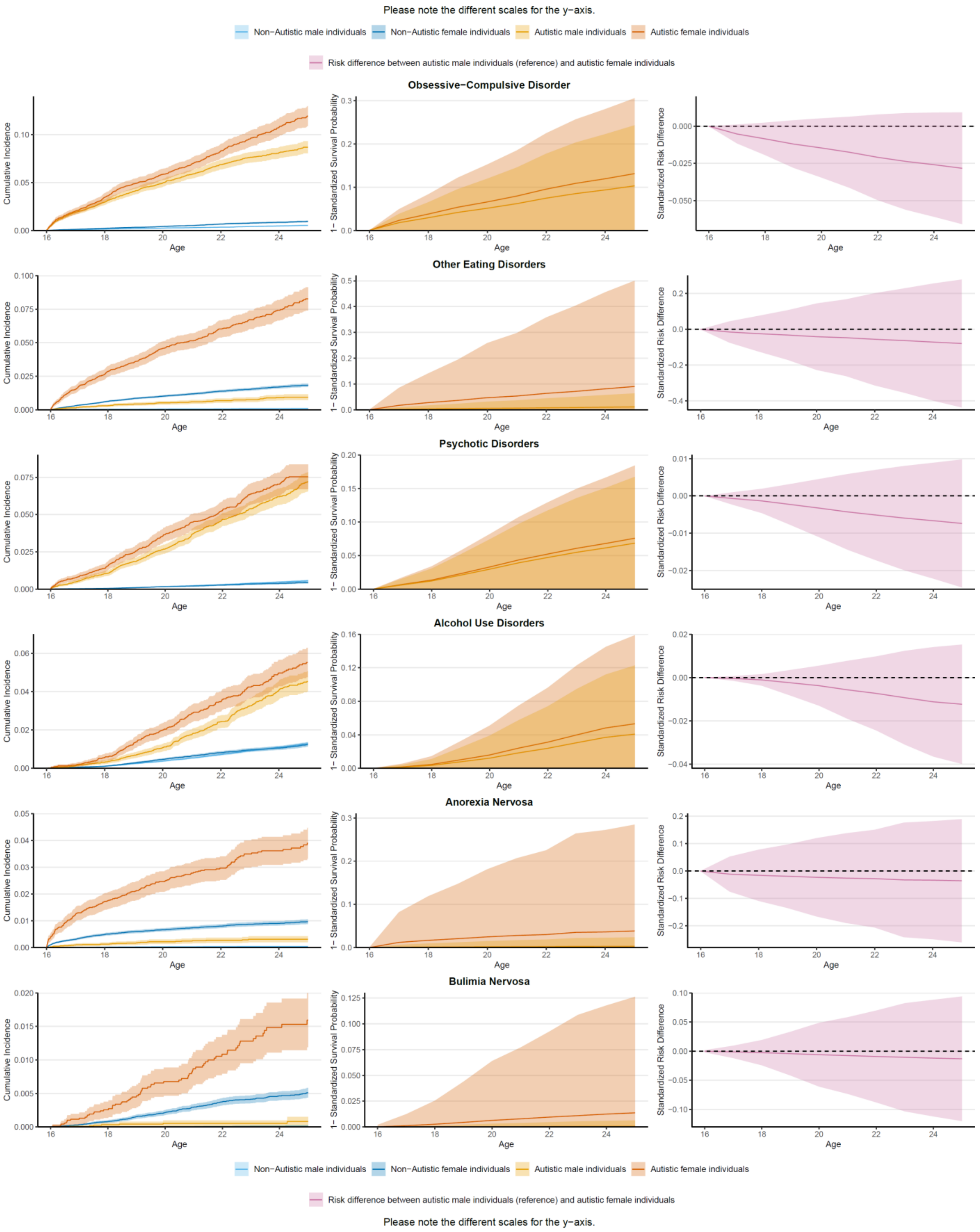

Note. The plots are ordered by the cumulative incidence of the disorders. The left graph shows the cumulative incidence for autistic and non-autistic women and men. The middle graph shows 1 – the birth year standardized survival probability for autistic women and men. The graph on the right shows the standardized risk difference between autistic women and men based on the standardized estimated in the middle graph.

© 2022 Martini M et al. *JAMA Psychiatry.*

eFigure 3. Cumulative Incidence, birth-year-standardized survival probability and risk difference for all diagnoses including only autistic individuals diagnosed before age 16.

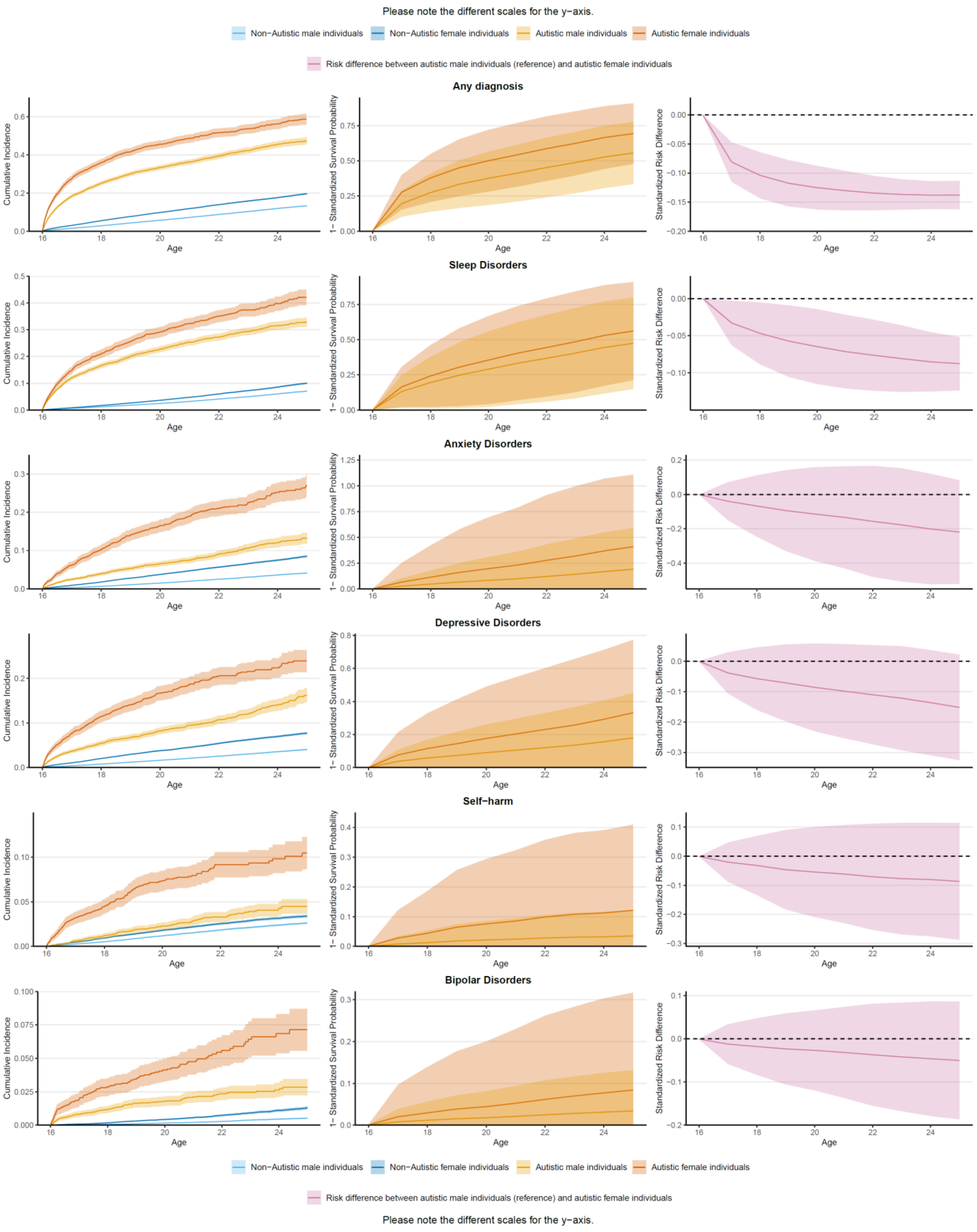

© 2022 Martini M et al. *JAMA Psychiatry.*

eFigure 3 continued.

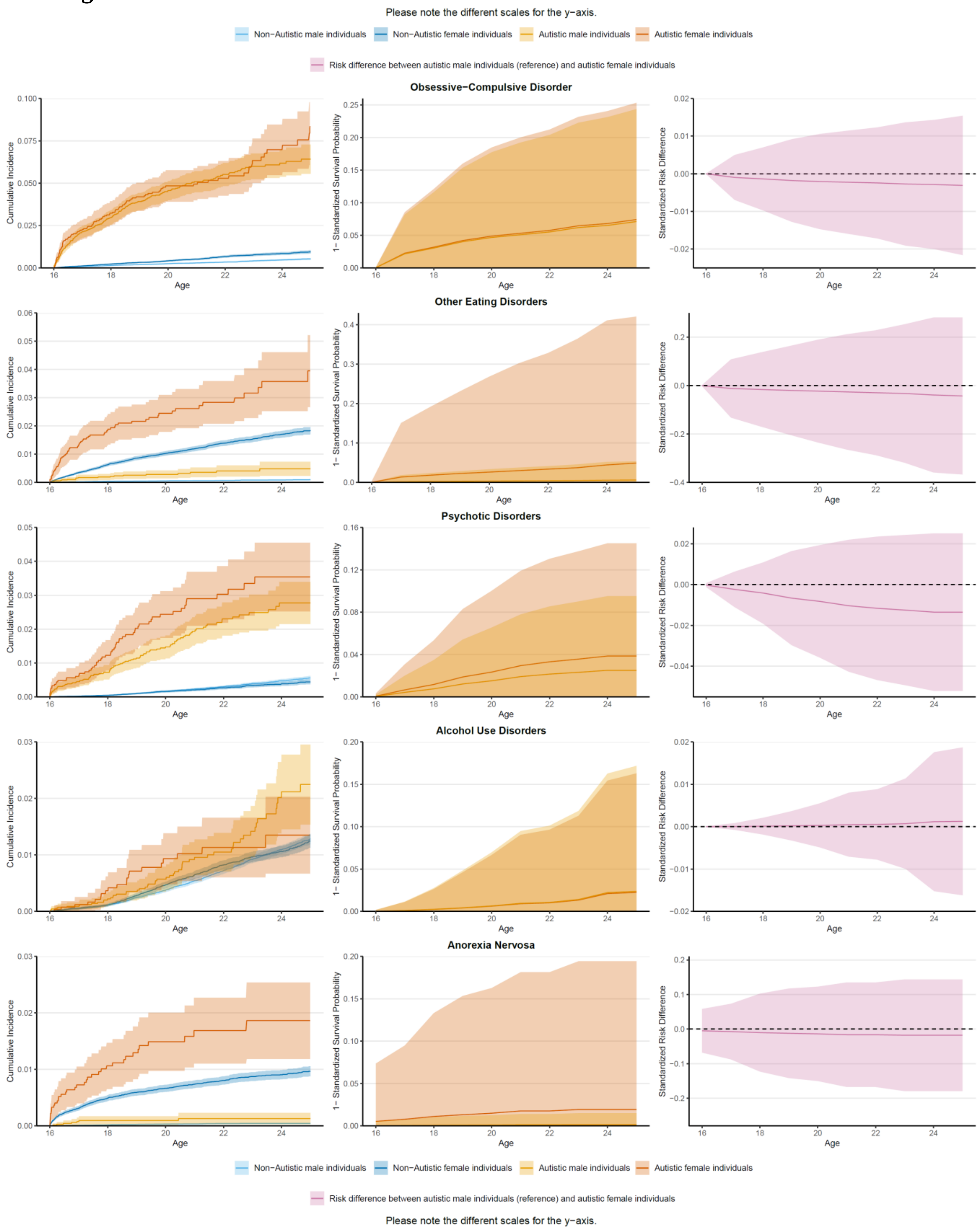

Note. The plots are ordered by the cumulative incidence of the disorders. The left graph shows the cumulative incidence for autistic and non-autistic women and men. The middle graph shows 1 – the birth year standardized survival probability for autistic women and men. The graph on the right shows the standardized risk difference between autistic women and men based on the standardized estimated in the middle graph. As there were no non-autistic males diagnosed with anorexia nervosa and other eating disorders for certain birth years, we had to combine birth years into categories (1985-1989,1990-1993,1994-1997) for these disorders. Bulimia nervosa is not shown in this graph, as the numbers among non-autistic males were too low.

© 2022 Martini M et al. *JAMA Psychiatry.*

eFigure 4. Cumulative Incidence, birth-year-standardized survival probability and risk difference for inpatient diagnoses.

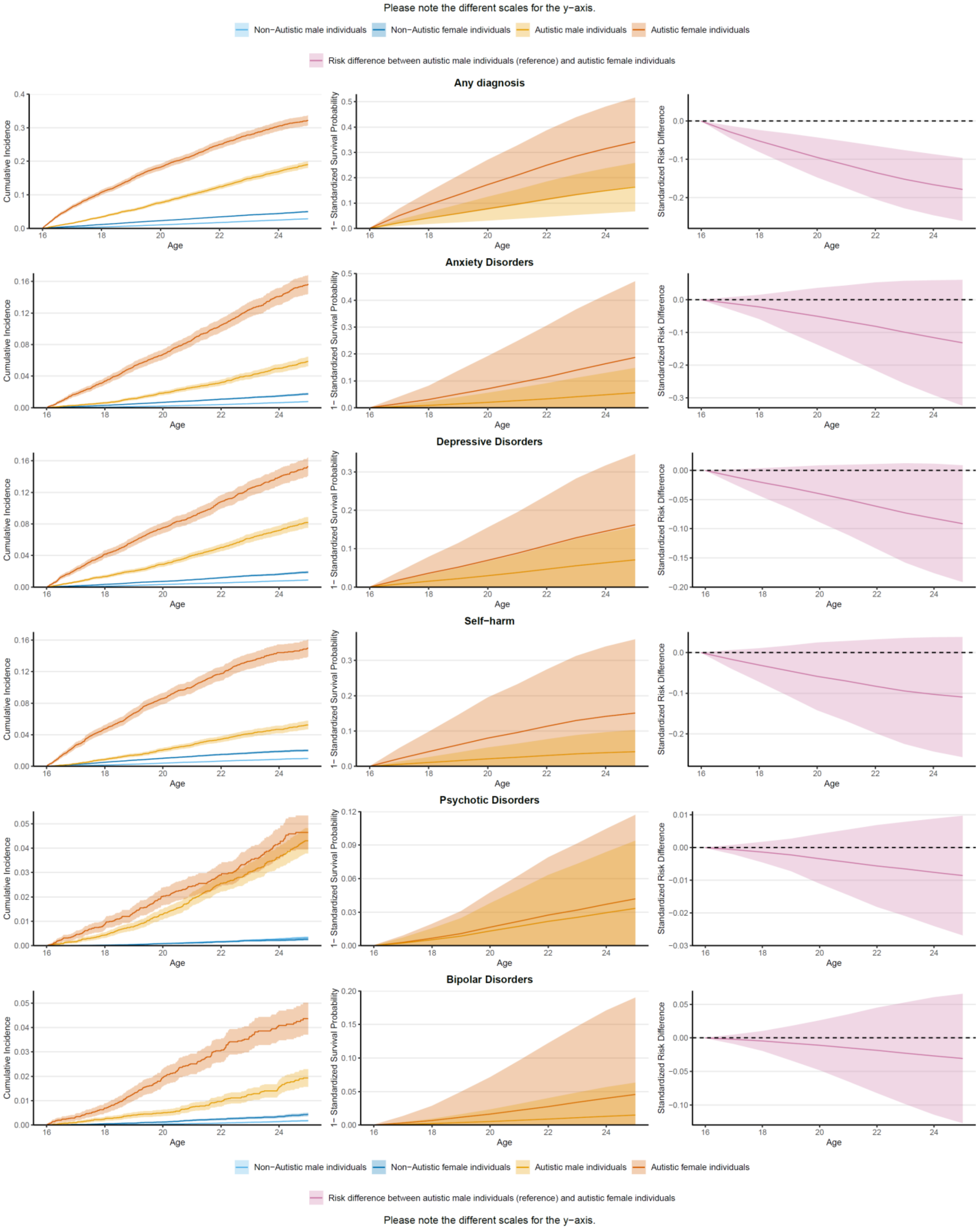

© 2022 Martini M et al. *JAMA Psychiatry.*

eFigure 4 continued.

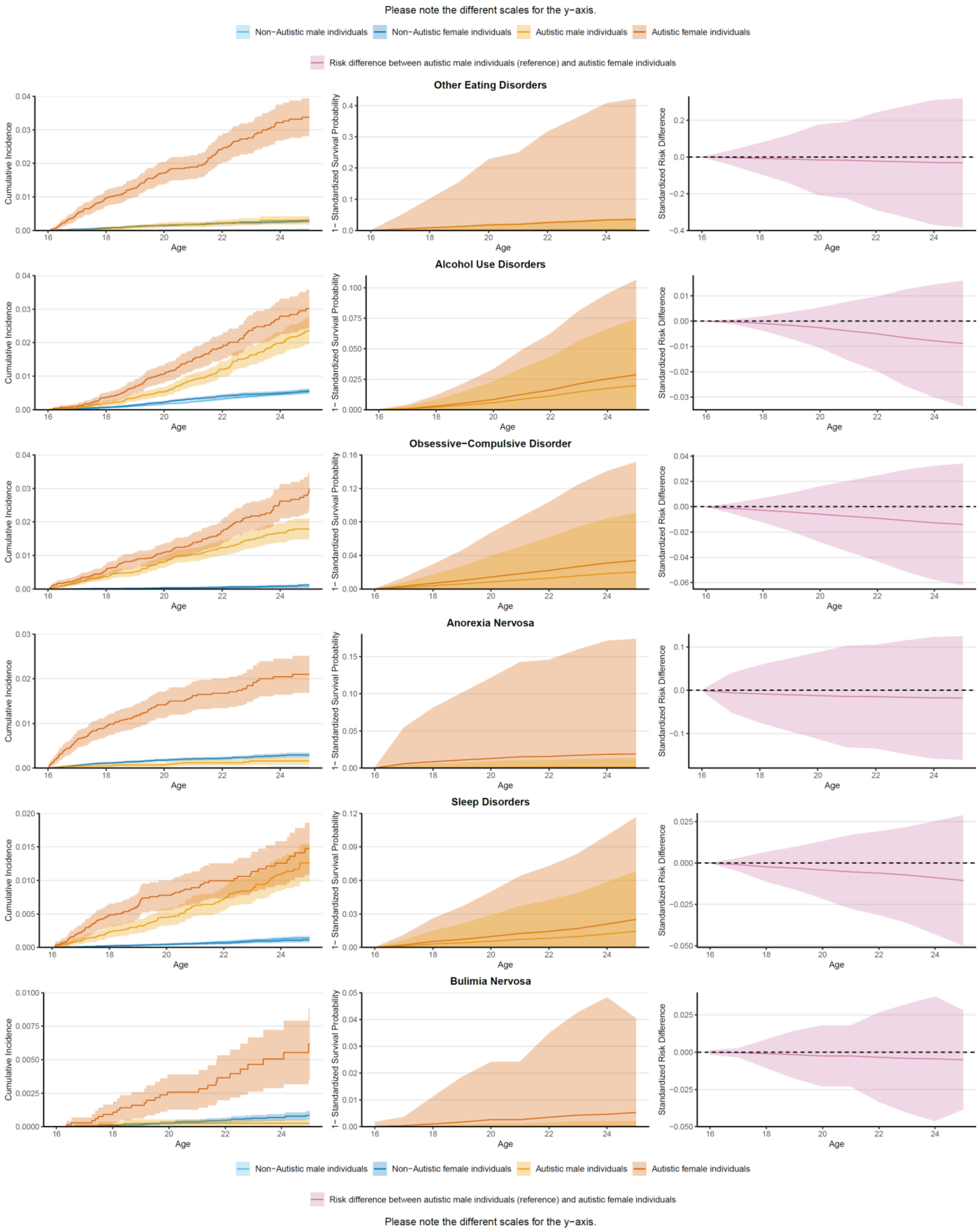

Note. The plots are ordered by the cumulative incidence of the disorders. The left graph shows the cumulative incidence for autistic and non-autistic women and men. The middle graph shows 1 – the birth year standardized survival probability for autistic women and men. The graph on the right shows the standardized risk difference between autistic women and men based on the standardized estimated in the middle graph.

© 2022 Martini M et al. *JAMA Psychiatry.*

eReferences

1. Axelsson O. The Swedish medical birth register. *Acta obstetricia et gynecologica Scandinavica*. 2003;82(6):491-492.
2. Ludvigsson JF, Almqvist C, Bonamy A-KE, et al. Registers of the Swedish total population and their use in medical research. *European journal of epidemiology*. 2016;31(2):125-136.
3. Ludvigsson JF, Andersson E, Ekbom A, et al. External review and validation of the Swedish national inpatient register. *BMC public health*. 2011;11(1):1-16.

© 2022 Martini M et al. *JAMA Psychiatry.*
